# The chromosome-scale genome of *Kobresia myosuroides* sheds light on karyotype evolution and recent diversification of a dominant herb group on the Qinghai-Tibet Plateau

**DOI:** 10.1093/dnares/dsac049

**Published:** 2022-12-12

**Authors:** Yu Ning, Yang Li, Shu Bin Dong, Hong Guo Yang, Chun Yi Li, Biao Xiong, Jun Yang, Yu Kun Hu, Xian Yun Mu, Xiao Fei Xia

**Affiliations:** Institute of Ecological Protection and Restoration, Chinese Academy of Forestry, Beijing, China; Institute of Wetland Research, Chinese Academy of Forestry, Beijing, China; Huzhou University, Huzhou, China; College of Biological Sciences and Technology, Beijing Forestry University, Beijing, China; Institute of Ecological Protection and Restoration, Chinese Academy of Forestry, Beijing, China; Institute of Wetland Research, Chinese Academy of Forestry, Beijing, China; Institute of Ecological Protection and Restoration, Chinese Academy of Forestry, Beijing, China; Institute of Wetland Research, Chinese Academy of Forestry, Beijing, China; College of Tea Science, Guizhou University, Guiyang, China; College of Biological Sciences and Technology, Beijing Forestry University, Beijing, China; Institute of Ecological Protection and Restoration, Chinese Academy of Forestry, Beijing, China; Institute of Wetland Research, Chinese Academy of Forestry, Beijing, China; College of Ecology and Nature Conservation, Beijing Forestry University, Beijing, China; Beijing Museum of Natural History, Beijing, China

**Keywords:** *Kobresia*, *Carex*, genome, karyotype evolution, alpine

## Abstract

*Kobresia* species are common in meadows on the Qinghai–Tibet Plateau. They are important food resources for local livestock, and serve a critical foundation for ecosystem integration. Genetic resources of *Kobresia* species are scarce. Here, we generated a chromosome-level genome assembly for *K. myosuroides* (Cyperaceae), using PacBio long-reads, Illumina short-reads, and Hi–C technology. The final assembly had a total size of 399.9 Mb with a contig N50 value of 11.9 Mb. The Hi–C result supported a 29 pseudomolecules model which was in consistent with cytological results. A total of 185.5 Mb (44.89% of the genome) transposable elements were detected, and 26,748 protein-coding genes were predicted. Comparative analysis revealed that *Kobresia* plants have experienced recent diversification events during the late Miocene to Pliocene. Karyotypes analysis indicated that the fission and fusion of chromosomes have been a major driver of speciation, which complied with the lack of whole-genome duplication (WGD) in *K. myosuroides* genome. Generally, this high-quality reference genome provides insights into the evolution of alpine sedges, and may be helpful to endemic forage improvement and alpine ecosystem preservation.

## Introduction

*Kobresia* plants comprise a species-rich lineage in the sedge family (Cyperaceae). In its natural form, *Kobresia* species dominate the meadows of the Qinghai-Tibet Plateau (QTP), playing a key role in providing feed for local livestock and maintaining the carbon balance in the world’s largest pastoral alpine ecosystem.^1,2^ With a broad distribution across the whole QTP, the *Kobresia* species complex (~40 species) provides an excellent model system for studying alpine evolution and mining special genetic resources.^3^ However, the molecular resources of this group are very limited, with the exception of a single chromosome-scale assembly of *Kobresia littledalei*.^4^ Recent studies have shown that sedges have a phenomenal range of chromosome number variation and unique ways of evolution.^5–7^ Sedge species have a tendency to speciate via fission and fusion of chromosomes because of the atypical features of their holocentric chromosomes.^8^; this might also mediate the evolution of homoploidy as reviewed by Wu *et al*.^9^ Most previous genetic studies of *Kobresia* plants rely on fragment-based methodologies, generating limited evidence for species delimitation and karyotype evolution.^2^ Thus, qualified genome assemblies are in urgent need to generate a comprehensive atlas of genetic elements in *Kobresia* taxa and clarify their evolutionary trajectories.

**Figure 1.**
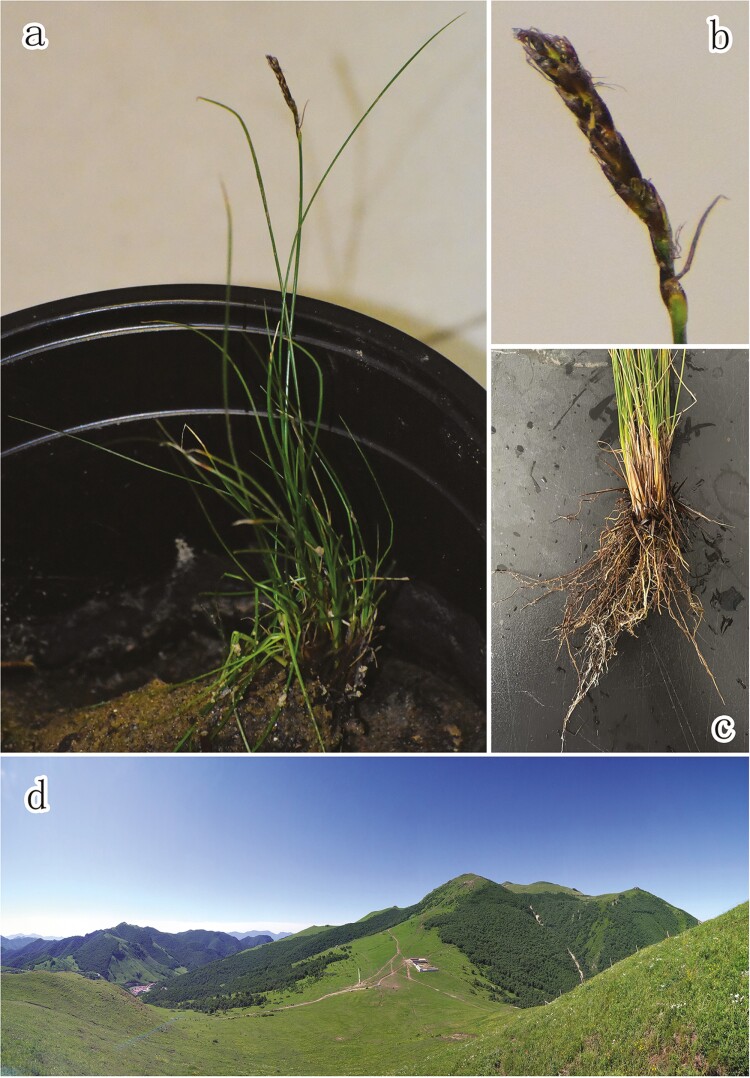
Images of *Kobresia myosuroides* and its habitat. (a) Whole plant; (b) mature inflorescence; (c) roots; and (d) habitat.

*Kobresia myosuroides* (Villars) Foiri^10^ is a critical sedge species in global alpine ecosystems (Fig. 1). This species engages in symbiotic relationships with ectomycorrhizal fungi, which mediates the transfer of amino acid nitrogen; this property of *K. myosuroides* indicates that it could be useful for the phytoremediation of alpine ecosystems.^11^ Unlike *K. littledalei*, which is restricted to southwestern China (Sichuan and Tibet) and prefers moist habitats,^4^*K. myosuroides* is widely distributed in the alpine environments in the northern hemisphere, including the QTP. Its ecological niche is also broad, from moist meadows to dry rocks and ridges, even under a canopy of shrubs.^10,12^ Previous studies about the origin of species diversity in the QTP have shown that, although the majority of diversification events happen *in situ*, the migrant and/or colonization processes also matter,^13,14^ especially for certain taxa, like sedges, with strong capability of long-distance dispersal.^15,16^ The migrant and/or colonization processes may be more important for the emergence of novel genetic resources, because the ‘isolation-recontact’ demographic processes tends to involve complex genetic elements dynamics, such as gene duplication and chromosome rearrangements. Thus, *K. myosuroides*, with a broad ecological niche and wide distribution, presents a good candidate for studies of the evolution and biogeography of *Kobresia* plants. Recent taxonomic treatment has incorporated the genus *Kobresia* into the genus *Carex* and a new nomenclature system has been established, according to which *Kobresia myosuroides* has been treated as a synonym of *Carex myosuroides*.^12^ To be consistent with the majority of previous literatures, we keep using *Kobresia myosuroides* hereafter in this paper.

To our knowledge, this assembly of *Kobresia myosuroides* represents the second published chromosome-scale assembly in the sedge family(Cyperaceae). The total size of the assembly was 399.99 Mb; it comprised 29 pseudo-chromosomes, and the contig N50 value was 11.99 Mb. We generated this assembly using circular consensus sequencing (CCS) on a PacBio platform, high-throughput chromosome conformation capture with Hi–C technology, and illumina sequencing. We also performed flow cytometry and fluorescence *in situ* hybridization (FISH) to confirm the results of the assembly. We then conducted genome annotation and comparative genomic analysis to clarify patterns of divergence among *Kobresia* species.

## Materials and methods

### Sample collection

We transferred vigorous individuals of *Kobresia myosuroides* from Dongling mountain (E115°30ʹ11ʹʹ, N40°3ʹ7ʹʹ) to a plantation at Beijing Forestry University. For genomic sequencing, fresh and healthy leaves were obtained. For transcriptomic sequencing, samples from four distinct tissues (stem, leaf, root, and spikelet) were collected. To exclude exogenous contaminants, all samples were collected with caution and stored at −80°C until extraction. The relevant specimen was deposited at the herbarium of Beijing Museum of Nature History (http://www.bmnh.org.cn/en/) under the voucher number BJM0271944 (contact xiaxiaofei@bmnh.org.cn).

### Genome sequencing

The CTAB (cetyltrimethylammonium bromide) approach was used to extract genomic DNA. A 15 kb DNA SMRTbell library was constructed following the standard protocol. This library was used for CCS on a PacBio Sequel platform. We also created a Hi-C library using HindIII enzyme following the methodologies recommended by BioMarker Technologies.^17,18^ Sequencing of the Hi–C library was established on Illumina NovaSeq 6000 platform.

### Transcriptome sequencing

For RNA-seq analysis, we acquired samples from four distinct tissues (stem, leaf, root, and spikelet). Paired-end libraries (average insertion size was approximately 350 bp) were generated and sequenced on an Illumina NovaSeq 6000 platform per the instructions provided by the Illumina standard mRNA-seq prep kit. The total volume of the outcome clean data is ~12.05 G (details in Supplementary Table S1).

### Genome survey and preliminary assembly

The genome survey was conducted using ~41.84 Gb (~108.39×) high-quality pair-end Illumina sequences (Supplementary Table S1). We utilized Genome Scope (v 2.0)^19^ and Jellyfish (v 2.1.4)^20^ to do the inspection. A k-mer distribution with a length of 19 nt generated from clean Illumina short-read data was chosen to determine the genome size, repeat content, and heterozygosity (Supplementary Fig. S1). We also carried out a flow cytometry to corroborate the obtained genome size (Supplementary Table S2, Fig. S3).

For PacBio long reads, we obtained a total volume of 21.74 Gb (~56×) CCS clean data through filtering and correcting of ~635.51 Gb raw data by employing the PBCCS pipeline with default settings (https://github.com/PacificBiosciences/ccs). The primary *de novo* assembly was established by utilizing the HIFIASM (v 0.14) pipeline.^21^ We further corrected the primary contigs using the Pilon (v1.24)^22^ with 41.84 Gb (~108.39× coverage) of illumina paired-end reads. Burrows-Wheeler Aligner (BWA, v 0.7.17)^23^ and SAMtools (v 1.13) (https://github.com/samtools/samtools/releases) were used to align the reads and convert SAM/BAM formats. We assessed the integrity and completeness of the obtained genome by implementing the BUSCO program (v 3.0)^24^ along with the embryophyta_odb10 database.

### Polishing and anchoring the preliminary assembly to chromosomes using Hi–C

In total, a volume of ~53.21Gb Hi–C clean data was obtained with trimming and filtering of raw reads. These data were truncated at the putative Hi–C junctions and aligned to the primary assembly with the BWA aligner. Only uniquely aligned pair reads with mapping quality higher than 20 were retained for further analysis. Invalid read pairs, including dangling-end, self-cycle, re-ligation, and dumped products, were filtered by HiC-Pro (v 2.8.1).^25^

For the Hi–C anchoring process, we first performed an error correction by dividing the preliminary scaffolds into equal partitions of ~50 kb volume. Then, the BWA algorithm was invoked to project the validated Hi–C data to these partitions. We kept only the singularly projected data and conveyed them to LACHESIS^26^ for further anchoring. Segment pairs showing inconsistency with the raw scaffolds were checked with caution. Erroneous placements and orientations exhibiting obvious discrete chromatin interaction patterns were manually treated. The outcome of Hi–C mapping was further validated using fluorescence *in situ* hybridization (FISH) with 4ʹ,6-diamidino-2-phenylindole (DAPI) staining^27^ and a telomere sequence probe (TTTAGGG)_6_^28^ (Supplementary Fig. S2).

### Protein-coding gene prediction

We utilized the MAKER pipeline^29^ to determine the high-quality protein-coding genes. Three methods were involved in the procedure: *de novo* gene predictions, transcript-based prediction, and homologous-based inference. Two *ab initio* gene-prediction software tools, Augustus and SNAP, were implemented to predict the *de novo* gene models. GeMoMa was employed to detect homology between target sequences and well-annotated genes from four species (*Triticum aestivum, Oryza sativa, Carex littledalei,* and *Panicum virgatum*). For the transcript-based prediction, RNA-sequencing data were mapped to the reference genome using Hisat and assembled by Stringtie. GeneMarkS-T and PASA software were used to predict genes based on the unigenes assembled by Trinity. Lastly, we integrated all the gene models of three approaches using the EVM software (v 1.1.1).^30^

### Functional annotation

Functional annotations of protein-coding genes of *K. myosuroides* were performed using BLASTP searches against the following six public databases with an *E*-value cut-off of 1.0 × 10^−5^: InterPro, eggNOG, Gene Ontology (GO), Cluster of Orthologous Groups of proteins (COG), Swiss-Prot and Kyoto Encyclopaedia of Genes and Genomes (KEGG). Protein domains were confirmed through querying against the InterPro and Pfam databases using InterProScan (v 5.1)^31^ and HMMER (v3.3).^32^ GO enrichment and KEGG pathway analysis were performed using the OmicShare online platform (https://www.omicshare.com/). The tRNAscan-SE (v 1.3.1)^33^ was employed to detect tRNA with eukaryote parameters. Barrnap (v 0.9)^34^ was utilized to identify rRNA genes. miRNAs were determined by searching miRBase (release 21) databases.INFERNAL1.1^35^ was employed to predict the snoRNA and snRNA genes with reference to the Rfam (release 12.0) database (details in Supplementary Table S6).

### Detection of repetitive elements

We jointly utilized homology-based and *de novo* approaches to detect the transposon elements (TEs) and tandem repeats. RepeatModeler2 (v2.0.1)^36^ was used to customize a *de novo* repeat library. Full-length long terminal repeat retrotransposons (fl-LTR-RTs) were identified using both LTRharvest (v1.5.9)and LTR_finder (v1.1). LTR_retriever (v2.8)^37^ was introduced to get the high-quality intact fl-LTR-RTs and non-redundant LTR library. Then, we built a non-redundant and species-specific TE library by combining the acquired *de novo* TE library with the extant databases: Repbase (v 19.06), REXdb (v3.0), and Dfam (v3.2). RepeatMasker v4.10^38^ was utilized to obtain the final TE sequences. For each LTR, the insertion time (*T*), determined as *T* = *K*/(2*r*) with a substitution rate (*r*) of 7 × 10^−9^, was also acquired. Meanwhile, we also computed tandem repeats using Tandem Repeats Finder^39^ and MIcroSAtellite identification tool (MISA v2.1)^40^ to improve the scope of repetitive elements detection (Supplementary Table S4).

### Phylogeny construction and gene family analyses

OrthoFinder (v2.4)^41^ was utilized to find the orthologous groups among *K. myosuroides* and eight reference species: *Oryza sativa*, *Kobresia littledalei*, *Phoenix dactylifera*, *Musa acuminata*, *Asparagus officinalis*, *Zostera marina*, *Bolboschoenus planiculmis*, and *Amborella trichopoda*. To minimize the redundancy stemming from alternative splicing, we kept only the longest transcripts of each gene model. Then, we chose the all-versus-all method to get the BLASTP alignments. Single-copy orthogroups were accordingly aligned and transformed to super-alignments through the concatenation method, with gap regions trimmed by Gblocks (v.0.91).^42^ Subsequently, these clean super-alignments were used to construct a ML phylogenetic tree by IQ-TREE (v 1.6.11)^43^ with a fitted model of JTT + F + I + G4 suggested by ModelFinder.^44^ The 1000 bootstrap analyses were implemented for each branch to test the robustness of inference.

We estimated the divergence time using MCMCTree in the PAML (v 4.9) package.^45^ The burn-in iterations were set as 5 × 10^5^. We retrieved the divergence time between *Amborella trichopoda* and *Oryza sativa* (173–199 Mya) from the TimeTree database^46^ as the calibrated molecular clock. To ensure the robustness of the results of the MCMCTree analyses, we performed all the related computations two times. CAFE (v 5.0)^47^ was utilized to detect the gene family contraction and expansion events. The family-wide *P*-value was calculated to verify the significance level of the contraction (expansion) events at specific branches, and the Viterbi *P*-value was used to test the validity of the contraction (expansion) events for each orthogroup. GO and KEGG enrichment analysis was then carried out using the ClusterProfile (v 4.0)^48^ pipeline for genes in the families with inferred expansion (contraction) events.

### Identification of positively selected genes(PSGs)

The CODEML module in PAML^45^ was used to identify the potential genes under positive selection. Specifically, OrthoFinder^41^ was used to obtain the single-copy gene families. The related codon sequences were generated through PAL2NAL. Based on the non-synonymous to synonymous substitution ratio, we chose the Branch-Site Model to infer the possible selection scenarios. The ancestral branch leading to the divergence of *K. myosuroides* and *K. littledalei* was set as the foreground branch. The statistical validity of Model A and Model A null was tested through a Chi-square method. We also implemented the Bayes Empirical Bayes (BEB) method to determine the posterior probabilities of the candidate genes. Genes with BEB score greater than 0.95 were finally considered as positively selected genes (PSG).

### Inference of synteny and potential whole-genome duplication (WGD) events

To investigate the potential whole-genome duplication (WGD) events in *K. myosuroides*, we extracted the candidate sequence pairs utilizing an all-versus-all search in BLASTP with an *E* value cut-off of 1.0 × 10^−8^. MCScanX^49^ was used to identify collinear blocks. Then synonymous substitution rates (Ks) of the collinear sequence pairs were calculated using wgd (v1.1.1).^50^ Combined with the result of Hi-C mapping, we visualized the gene density, GC content, repeat content, and collinearity on individual pseudochromosomes using the Circlize package (https://cran.r-project.org/web/packages/circlize/index.html).

## Results and discussion

### Evaluation of the genome assembly

**Figure 2.**
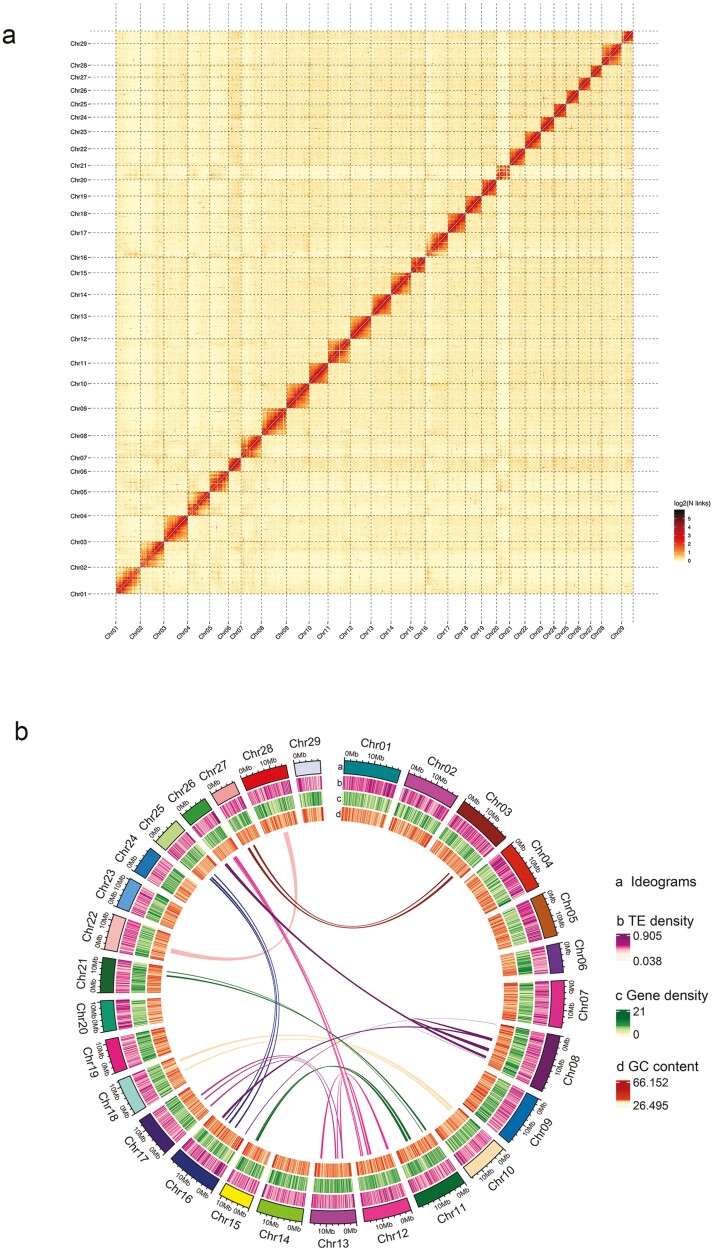
Overview of the *Kobresia myosuroides* genome assembly. (a) Heatmap showing Hi–C interactions at a resolution of 300 kb. The changes in colour from yellow to red indicate the frequencies of Hi–C links alter from low to high and (b) intra-genome synteny and distribution of genomic features. The four circular tiers represent chromosome ideograms, TE density, gene density, and GC content respectively (See online version for colour figure).

Our chromosome-scale genome assembly of *K. myosuroides* is only the second such assembly for any member of the family Cyperaceae. A total volume of 21.74 Gb (~56×) of Pacbio CCS long reads data, together with 53.21 Gb (~133×) of Hi-C data, and 41.84 Gb (~108.39×) of Illumina data (Supplementary Table S1) were generated to facilitate the assembly and annotation. Specifically, we construct the preliminary *de novo* assembly using Pacbio CCS long reads, consisting of 220 contigs with a total length of 413.15 Mb (Supplementary Table S3). With the integration of Hi–C data, this preliminary assembly was then fitted to a model of 29 pseudochromosomes (Fig. 2a, details in Supplementary Fig. S4 and Table S8). Approximately 96.82% of the *de novo* assembly was properly anchored and orientated. The total contig length of the final genome assembly is 399.99 Mb, and the N50 values for the contigs and scaffolds were 11.99 Mb and 15.45 Mb, respectively; the mapping rates and sequencing coverage scores were also consistent (details in Supplementary Table S7).

The high quality of our assembly of *K. myosuroides* are supported by: (i) the new assembly makes a considerable improvement relative to the extant genome assembly of sedge plants. The genome of *Kobresia littledalei* provided by Can *et al*.^4^ represents the first published chromosome-scale genome of sedge species (two draft genomes of *K. pgymea* and *K. roylena*^51^ have also been published). The genome size of *K. myosuroides* is similar to that of *K. littledalei* (399.99 Mb vs. 373.85 Mb, respectively). The completeness and continuity of our *K. myosuroides* assembly are higher, as indicated by the higher N50 values at both the contig (approximately five times higher) and scaffold (approximately six times higher) scale. The BUSCO scores also support the better performance of *K. myosuroides* assembly (overall 95.04% vs. 84.50%, details in Table 1). The gap rate of the final assembled *K. myosuroides* sequences was estimated to be 0.001% (4100 Ns), which is similar to gap rates reported for recent genome assemblies of alpine herbs endemic to the QTP.^52^ (ii) Our assembly has strong support from empirical data. Our kmer-based genome survey showed an approximate genome size of 386 Mb. The final assembly of *K. myosuroides* has a similar size of ~400 Mb, which is highly supported by the result of flow cytometry experiment (~407 Mb genome size, Supplementary Table S2). The outcomes of fluorescence *in situ* hybridization (FISH) showed that the basic chromosome number of *K. myosuroides* was 29 and the ploidy level was 2× (Supplementary Fig S2). This result is also consistent with the Hi–C interaction matrices displaying a diagonal pattern, consisting of 29 blocks for the intra-chromosomal interactions in all pseudochromosomes (Fig. 2a). Based on these evidences, it is reasonable to treat this new assembly of *K. myosuroides* with high confidence.

**Table 1.** Statistics of the final assembly of *Kobresia myosuroides* with reference to the only available chromosome-scale sedge genome assembly (*K. littledalei*)

Species	*Kobresia myosuroides*	*Kobresia littledalei*
Sequence		
Assembly size (bp)	399,997,527	373,852,675
GC content (%)	36.16	35.44
Number of scaffolds	225	523
Longest scaffold (bp)	19,428,065	7,550,132
Scaffold N50 size (bp)	15,447,987	2,548,827
Number of contigs	266	1,210
Longest contig (bp)	18,885,280	NA
Contig N50 size (bp)	11,995,645	2,253,412
Pseudochromosome		
Number	29	29
Anchored rate (%)	96.82	96.28
Size range (M)	8.55–19.43	2.46–19.67
BUSCO score		
Complete BUSCOs (%)	95.04	84.50
Complete and single-copy BUSCOs (%)	91.82	75.90
Complete and duplicated BUSCOs (%)	3.22	8.60
Fragmented BUSCOs (%)	1.30	2.30
Missing BUSCOs (%)	3.66	13.20
Total groups searched	1,614	1,440

NA: not available.

### Genome annotation and gene features

Repetitive sequences comprised 51.89% (approximately 214.39 Mb) of the *K. myosuroides* genome. 86.51% (~185.45Mb) of the total repetitive sequences are transposable elements (TEs), accounting for 44.89% of the total genome (details in Supplementary Table S5).TEs comprise a major fraction of many plant genomes and are critically important regulatory elements that affect adaptive traits in alpine environments, such as thermal sensing, reproduction through selfing, and oxidative responses.^53,54^ However, the proportion of long terminal repeat retrotransposons (LTR-RTs), 21.68% of the genome, was considerably smaller than that for several previously published plant genomes, such as 43.6% for caper spurge (*Euphorbia lathyris*),^55^ 47.08% for *Strobilanthes cusia*.^56^ Moreover, a consistent tendency for less proportion of LTR-RTs is possible in alpine sedges, for example, 27.87% (*K. littledalei*),^4^ 25.47% (*Carex parvula*), and 23.84% (*Carex kokanica*).^51^ The reason for this pattern needs further investigation.

**Figure 3.**
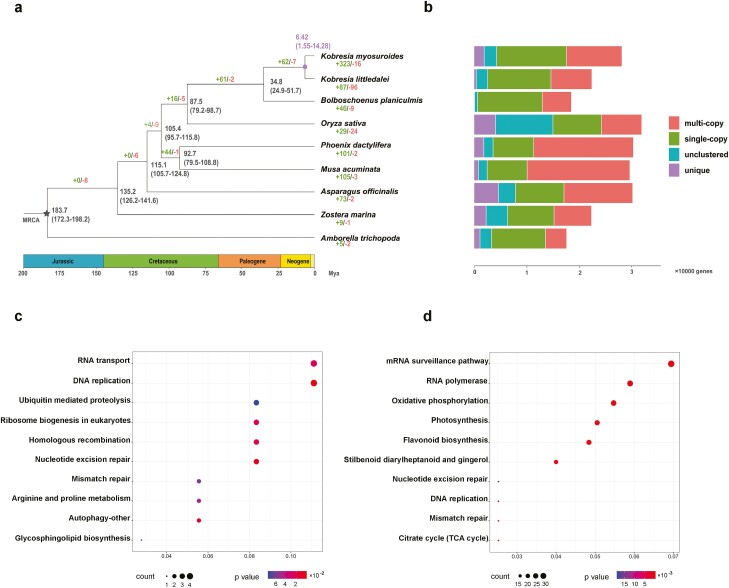
Phylogenetic and enrichment analysis of the *Kobresia myosuroides* genome. (a) Phylogenetic tree based on 1,175 single-copy orthologs and rooted using *Amborella trichopoda* as the outgroup. Number of expansions is denoted in green, and the number of contractions is indicated in red. Numbers at nodes show the inferred divergence times with 95% confidence intervals; (b) copy number distribution in the *K. myosuroides* genome and the other eight plant genomes used in the phylogenetic analysis. Results of the enrichment analysis of (c) positively selected genes and (d) unique genes with projections onto the KEGG pathways in the *K. myosuroides* genome. The size and colour of the symbol are scaled to gene counts and the significance level, respectively (See online version for colour figure).

Based on the repeat-masked genome, we predicted 26,748 protein-coding genes for *K. myosuroides* (Table 2). Totally, 95.42% (25,524) of the predicted genes were successfully matched with a function annotation in at least one public database (exampled in Table 3), and this percentage is similar to that for the *K. littledalei* genome (22,892 genes from 23,136 candidates pool, 98.95% annotation ratio). These two species also have similar numbers of genes, average gene lengths, and average numbers of exons (Supplementary Fig. S5). Gene function analysis highlights the importance of expression regulation and flavonoid metabolism in the evolution of *K. myosuroides*. Totally, we identified 439 gene families that were unique to *K. myosuroides* (Supplementary Fig. S6). We also found 323 gene families that have undergone expansions and 16 gene families that have undergone contractions in the lineage leading to *K. myosuroides* following its divergence from *K. littledalei* about 6.42 Mya ago(Fig. 3a). Results of the pathway analysis (Fig. 3c) show that selection favours robust replication mechanisms, and this is supported by the high proportions of genes involved in ‘DNA replication’, ‘homologous recombination’, and ‘ubiquitin-mediated proteolysis’. Furthermore, RNA-related regulatory processes might play a key role in mediating the adaptation of *K. myosuroides* to alpine habitats, as indicated by the high proportions of genes involved in ‘mRNA surveillance’, ‘RNA polymerase’, and ‘RNA transport’. Liu *et al.*^57^ noted that alleles involved in the regulation of gene expression might play a more important role than coding sequences in adaptation and speciation in alpine environments. Our findings are consistent with this hypothesis.

**Table 2.** Summary of the gene prediction and annotation results of *Kobresia myosuroides* and *K. littledalei*

Species	*Kobresia myosuroides*	*Kobresia littledalei*
*Gene Prediction*		
Number of predicted genes	26,748	23,136
Total coding sequence length (bp)	101,678,764	81,810,189
Mean gene length (bp)	3,801.36	3,545.25
Mean CDS length (bp)	1,282.69	1,163.41
Mean number of exons per gene	5.55	5.39
Mean number of introns per gene	4.55	NA
*Gene Annotation*		
NR Annotated Percent (%)	92.36	94.92
Swiss-Prot Annotated Percent (%)	75.04	77.03
KEGG annotated percent (%)	67.98	71.82
Pfam annotated percent (%)	81	76.23
GO Annotated percent (%)	78.18	69.68
Total account of annotated genes	25,524	22,892
Total annotated percent (%)	95.42	98.95

NA: not available.

**Table 3.** Information on candidate genes in the *K. myosuroides* genome involved in adaptation to alpine habitat

Entry name	EntryID	Candidate genes	Short description
ANR_GINBI	Q5XLY0	*Kmy02G003000, Kmy02G003020, Kmy03G005560 etc.*	Key enzyme for the biosynthesis of proanthocyanidins downstream of the flavonoid metabolism pathway
HSP70_SOYBN	P26413	*Kmy01G004350, Kmy05G006140, Kmy08G003570 etc.*	Protecting plants against abiotic stresses, including salt, drought, heat, and cold stress
MYB_AVIMB	P01104	*Kmy24G004060, Kmy24G004080, Kmy24G004160*	Regulating various processes such as responses to stress, development, and metabolism.
P21_SOYBN	P25096	*Kmy05G006260, Kmy10G005960, Kmy10G005970*	DNA repair, transcriptional regulation, and modulation or inhibition of apoptosis
ICE1_ARATH	Q9LSE2	*Kmy05G003180, Kmy28G006850*	Tolerance of chilling and freezing in plants
NAC2_CHLRE	Q9LEM8	*Kmy24G001160*	Mainly involved in plant growth, development, and biotic or abiotic stress responses
MSH1_ARATH	Q84LK0	*Kmy25G001850*	Repairing DNA mismatches and correcting insertion-deletion loops
RLK7_ARATH	F4I2N7	*Kmy03G000560, Kmy05G000610, Kmy05G000960 etc.*	An important transcription factor that regulates resistance to abiotic stress in plants
JHS1_ARATH	A0A1P8	*Kmy03G010960*	Key enzyme involved in DNA replication and damage repair, as well as shoot apical meristem development

The detailed full list is shown in Supplementary Table S9.

### Speciation and karyotype evolution

**Figure 4.**
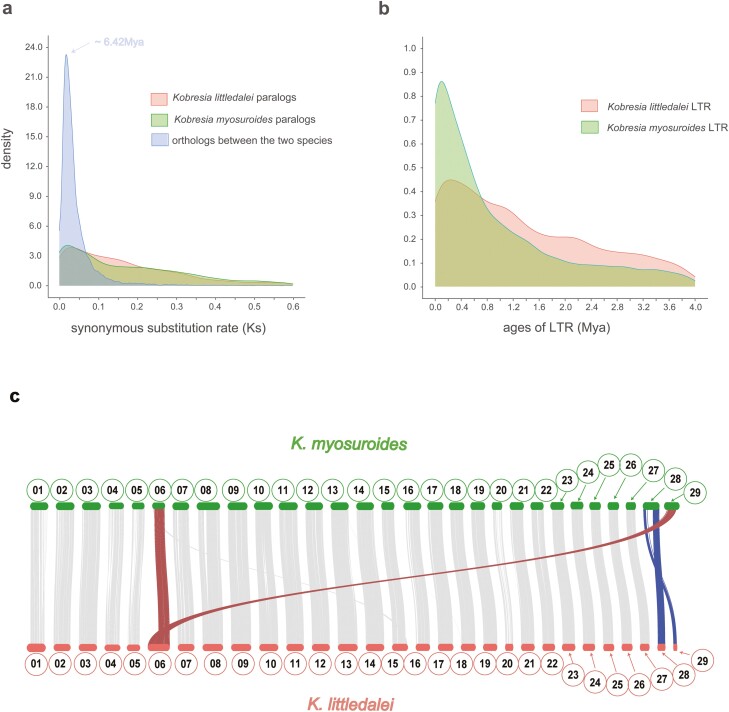
Inter-genomes collinearity and rates of evolution of *Kobresia myosuroides* and *Kobresia littledalei*. (a) Synonymous substitution rate distribution; (b) LTR insertion time distribution; and (c) inter-genome collinearity at the whole-genome scale. Chromosome IDs are shown inside circles. Signals for chromosome fusion and fission are highlighted with colours (See online version for colour figure).

A fully supported phylogeny was constructed using 1,175 single-copy genes from nine species in the main monocot clades (Fig. 3a and b). Our findings indicate that *K. myosuroides* and *K. littledalei* diverged approximately 6.42 Mya, which is similar to the divergence time between *K. royleana* and *K. littledalei* (approximately 6.2 Mya). *K. pygmea* and *K. littledalei* diverged later (approximately 5.0 Mya).^4,51^ Although the origin of *Kobresia* is assumed to be in the early Miocene (approximately 20 Mya),^58^ they might have rapidly diversified during the late Miocene (23–5.3 Mya) to the Pliocene (5.3–2.6 Mya), a geological period thought to be critical for the speciation of *Carex* taxa^59^. Abundant uplift events have occurred in the QTP from the Miocene to the Pliocene, and rapid *in situ* diversification has been observed in several alpine lineages since the late Miocene.^59–61^ The timing of *Kobresia* speciation is consistent with this general pattern. In addition, the transposition of most LTRs has occurred recently in both species (Fig. 4b). Most LTR components of these two species have emerged following the speciation event (<1.5 Mya). This rapid increase in the emergence of LTR components might reflect the effects of climatic oscillations and habitat dynamics during Pleistocene glacial cycles, as has been observed in other alpine plants that have recently diversified.^62–64^ This emphasizes the importance of regulatory genes in alpine adaptation and indicates the evolution of the *Kobresia* plants may still be ongoing.

The karyotype of *K. myosuroides* provides more detailed information on the speciation process. Fission and fusion are the main mechanisms of chromosome evolution in sedges, and this contrasts with the prevalence of chromosome duplication in other angiosperm clades.^7^ The collinearity between *K. myosuroides* and *K. littledalei* at the whole-genome scale (Fig. 4c) shows that: (1) most collinear orthologs between these two species have a 1:1 ratio, suggesting no lineage specific WGD for both species. Furthermore, both the *K. littledalei* and *K. myosuroides* present no substantial paralogs blocks in the results of within-genome collinearity analysis (Supplementary Fig. S8). Previous study has shown that apparent blocks of colinear segments within a specific genome were the strongest evidence of WGD events,^50^ so it is likely that WGD events do not act as the main driven force for *Kobresia* speciation; (2) the fission and fusion of chromosomes have likely occurred. Specifically, a major portion of *K. myosuroides* CHR 06 is combined with *K. myosuroides* CHR 29 to form *K. littledalei* CHR 06. In addition, *K. myosuroides* CHR 28 comprises *K. littledalei* CHR 28 and CHR 29, but with the loss of an intermediate section (Fig. 4c, details in Supplementary Fig S9). The high gene density of *K. littledalei* CHR 06 supports our hypothesized mechanism of chromosome formation, given that the abundance of genes in its assumed origin (*K. myosuroides* CHR 29 and CHR 06 are similar (Fig. 2b). Holocentric chromosomes with diffuse centromeres might facilitate fission and fusion; however, fission and fusion might be more difficult in chromosomes with fixed centromeres.^6,8^ Our FISH results also confirmed that *K. myosuroides* possesses holocentric chromosomes (Supplementary Fig. S2). Generally, our genome assembly provides insights into karyotype evolution at whole-genome scale. Our data support the absence of WGD for these two Kobresia species. Chromosome fission and fusion may have played a key role in the speciation process.

## Supplementary Material

dsac049_suppl_Supplementary_Material

## Accession numbers

The GenBank accession number for this reported genome assembly and the raw reads is PRJNA860309. The related BioSample accession number is SAMN29837785, including the RNA-Seq data involved in this paper.
