# Supplementary material for: The chromosome-scale genome of Kobresia myosuroides sheds light on karyotype evolution and recent diversification of a dominant herb group on the Qinghai-Tibet Plateau
Source: DNA Res. 2022 Dec 12;30(1):dsac049. doi: 10.1093/dnares/dsac049 (PMC9835760; doi:10.1093/dnares/dsac049)
Supplement: dsac049_suppl_Supplementary_Material [file dsac049_suppl_supplementary_material.docx]

|  |
| --- |

**Supplementary materials for “The chromosome-scale genome of *Kobresia myosuroides* provides insights into the karyotype evolution and recent diversification of a dominant herb group in the Qinghai-Tibet Plateau”**

NING,Yu^1,2^; LI,Yang^3^; DONG, Shu Bing^4^; YANG, Hong Guo^1,2^; XIONG, Biao^5^; YANG, Jun^4^; HU, Yu Kun^1,2^; MU, Xian Yun^6^; XIA, Xiao Fei^*7^

*1:* *Institute of Ecological Protection and Restoration, Chinese Academy of Forestry, Beijing, China;*

*2: Institute of Wetland Research, Chinese Academy of Forestry, Beijing, China;*

*3:* *Huzhou University, Huzhou, China;*

*4: College of Biological Sciences and Technology, Beijing Forestry University, Beijing, China;*

*5: College of Tea Science, Guizhou University, Guiyang, China*

*6: College of Ecology and Nature Conservation, Beijing Forestry University, Beijing, China;*

*7: Beijing Museum of Natural History, Beijing, China*

^*^Corresponding author. Email: xiaxiaofei@bmnh.org.cn


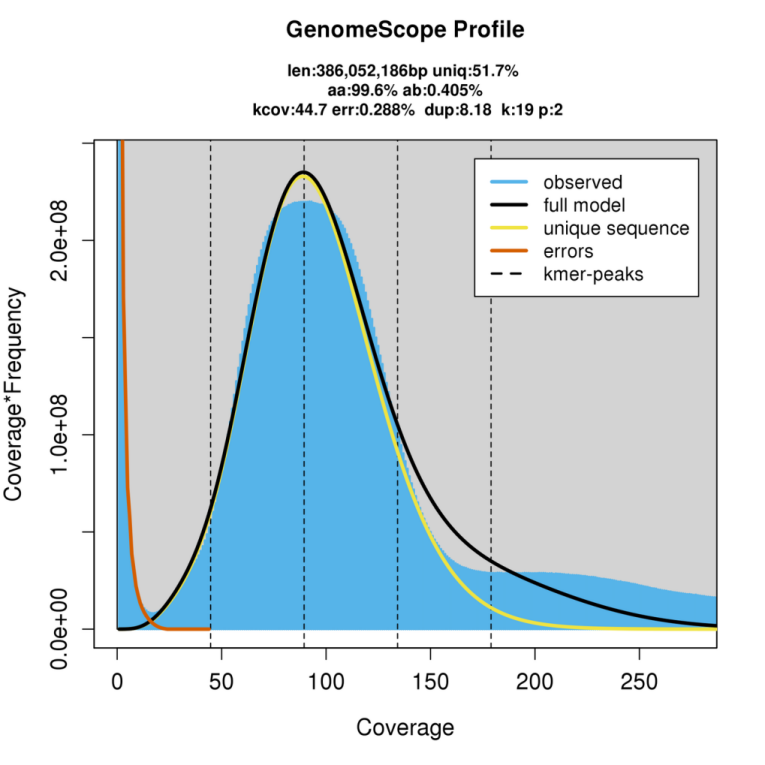


**Supplementary Figure S1. The *K*-mer analysis used to evaluate** ***Kobresia myosuroides* genome.** The frequency of 19-mers were shown.The genome size of *K. myosuroides* was estimated to be ~ 386 Mb, with an approximate repetitive content of 48.25% and heterozygosity of 0.40%. The putative ploidy level is 2X.

**
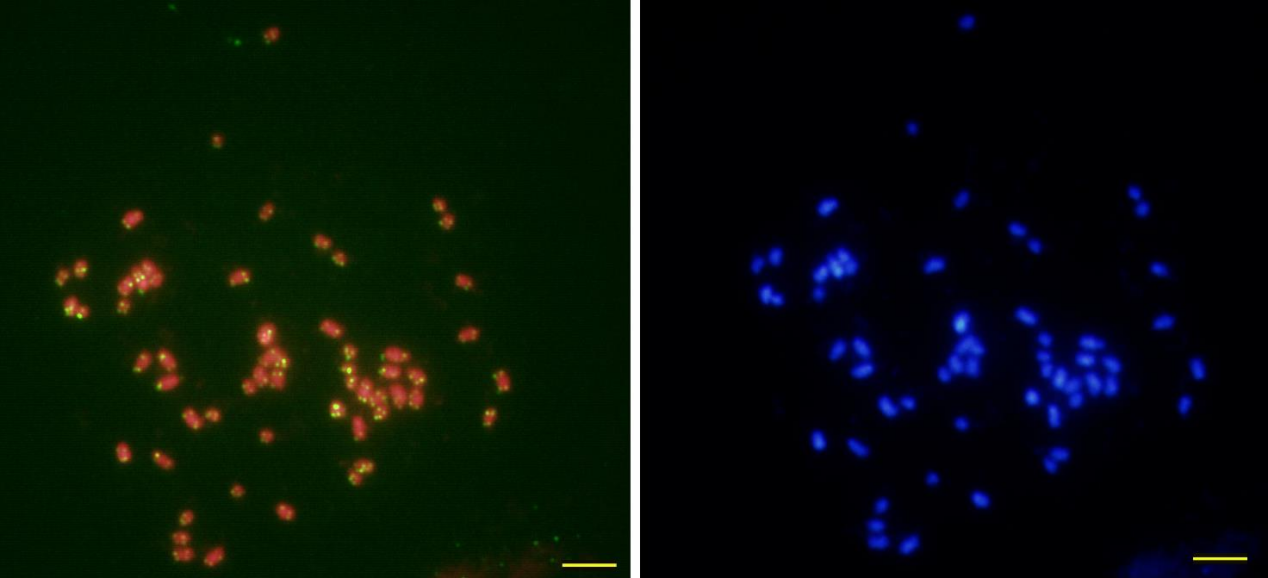
**

**c**

**b**

**a**

| 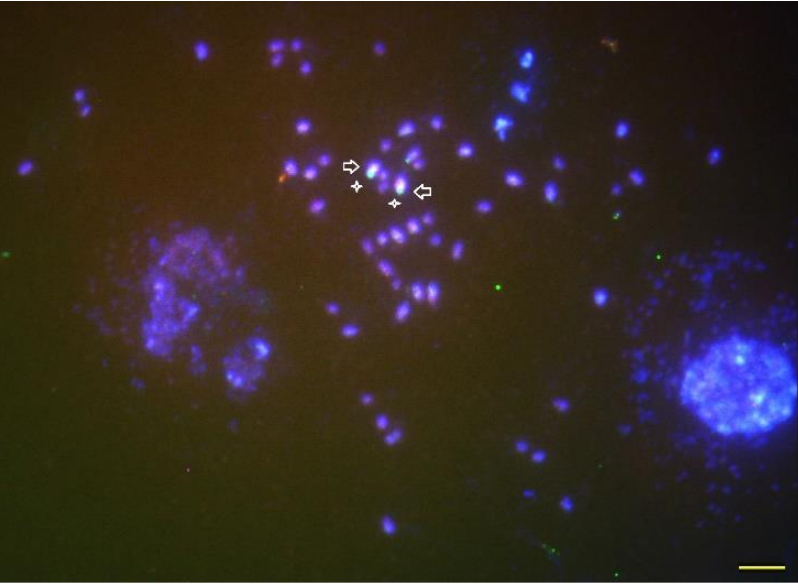 |
| --- |

**Supplementary Figure S2. The result of Fluorescence in situ hybridization (FISH)** Both (a) hybridization using telomere sequence probe (TTTAGGG)_6_ and (b) DAPI staining show a consensus chromosome number. (c) Results of hybridization using 5SrDNA probe(red) and 18SrDNA probe(green). The arrows and aterisks point to the two chromosomes showing signals

| 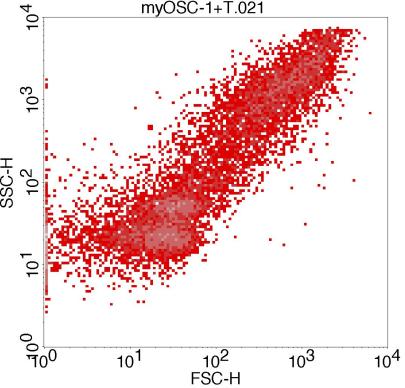 | 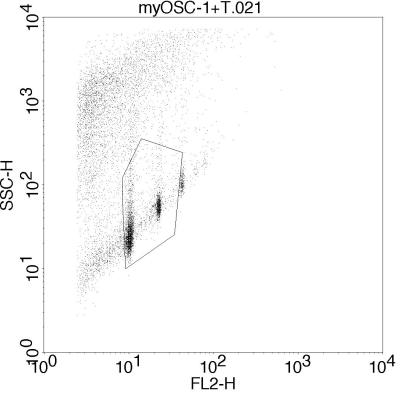 | 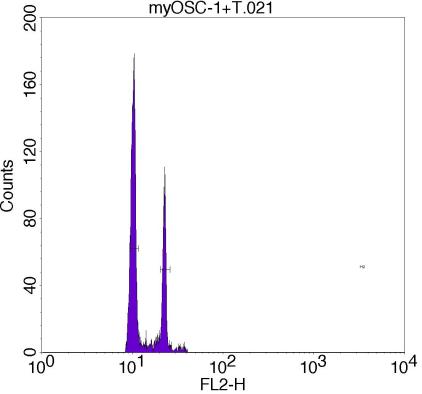 |
| --- | --- | --- |
| 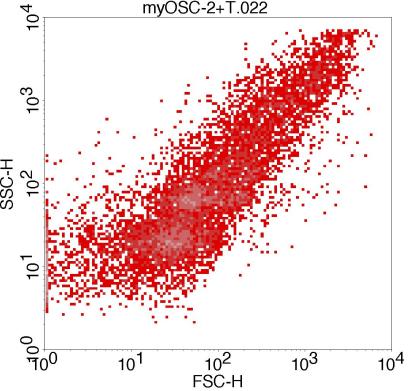 | 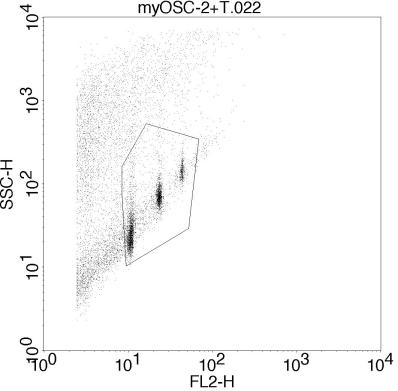 | 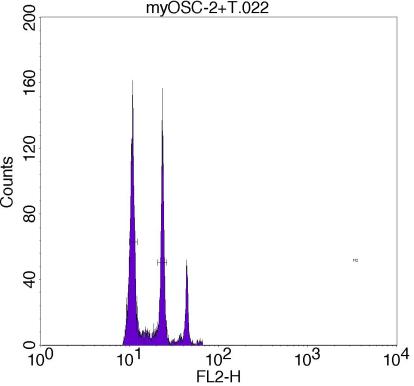 |
| 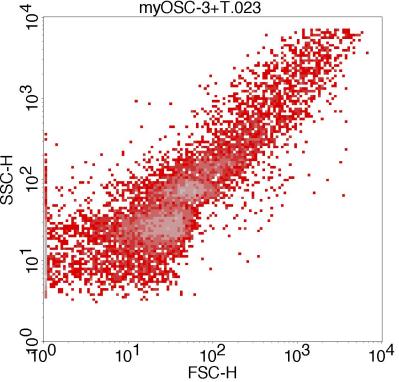 | 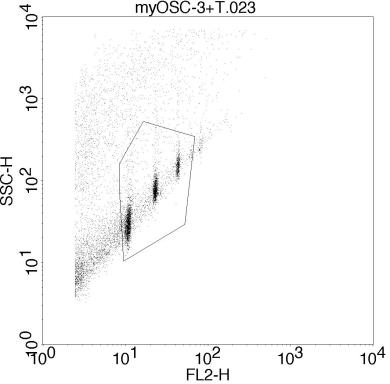 | 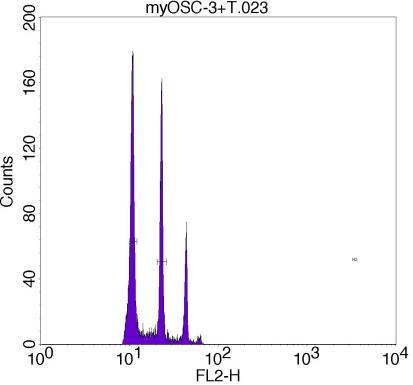 |

**Supplementary Figure S3. The result of flow cytometry of Kobresia myosuroides using tomato genome (880Mb) as reference.** Detailed value are shown in supplementary table2

| 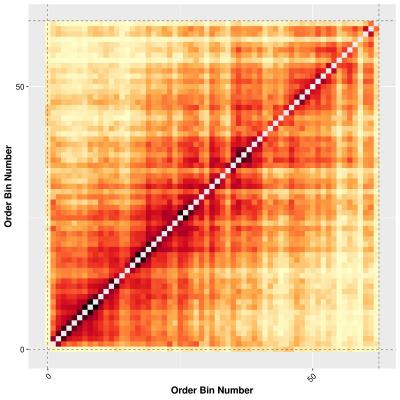  Chr01 | 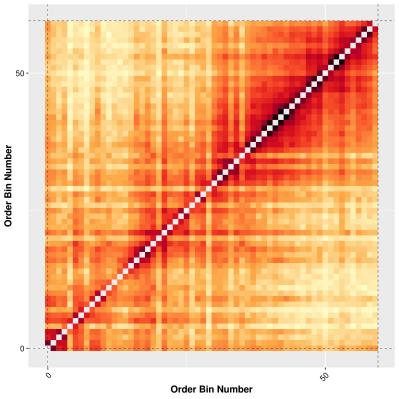  Chr02 | 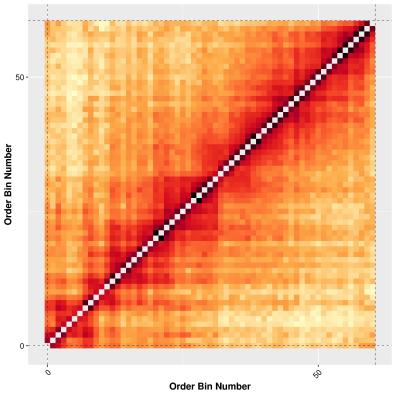  Chr03 |
| --- | --- | --- |
| 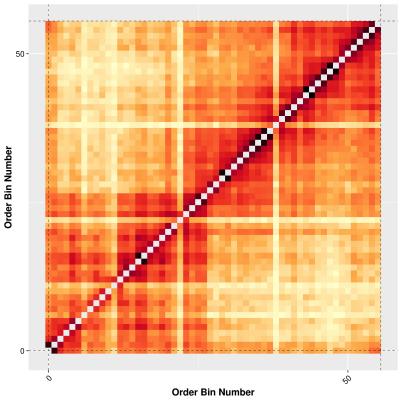  Chr04 | 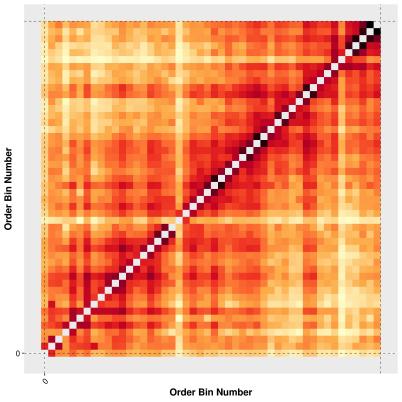  Chr05 | 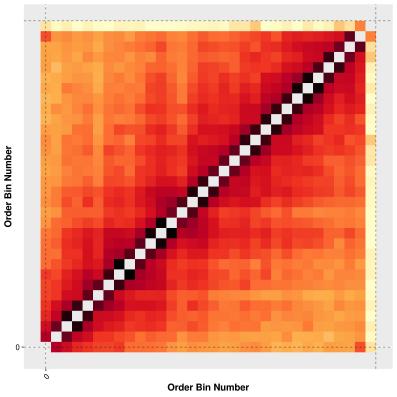  Chr06 |
| 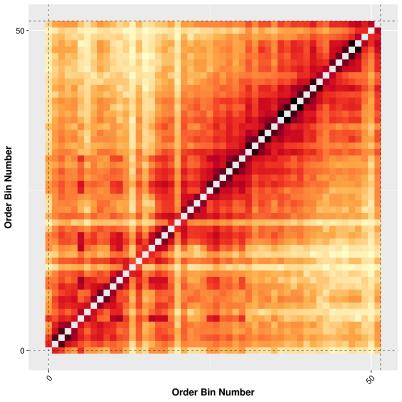  Chr07 | 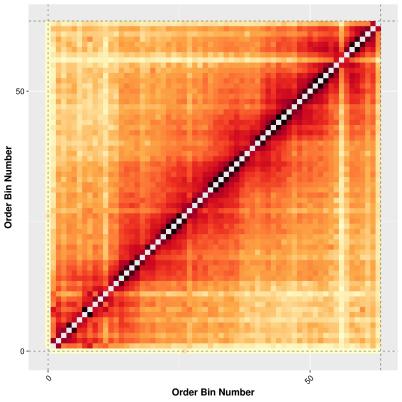  Chr08 | 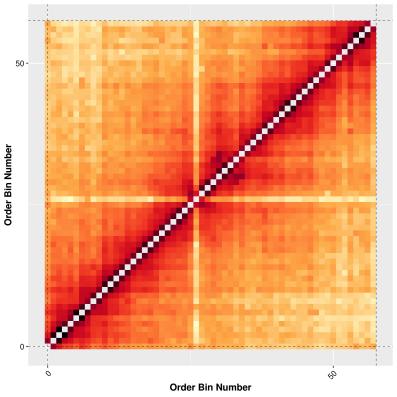  Chr09 |
| 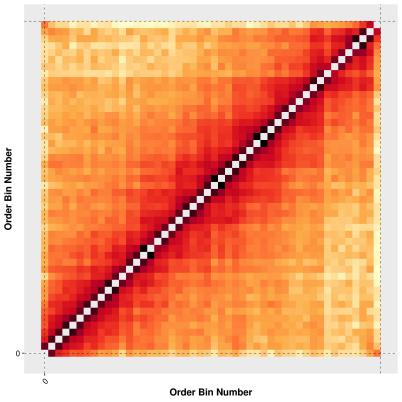  Chr10 | 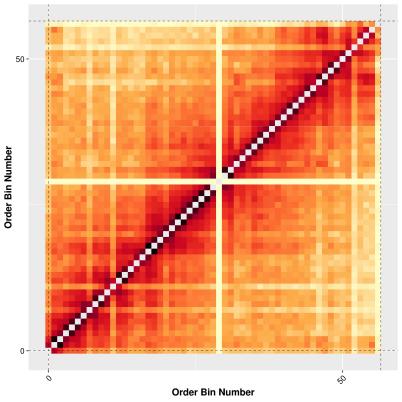  Chr11 | 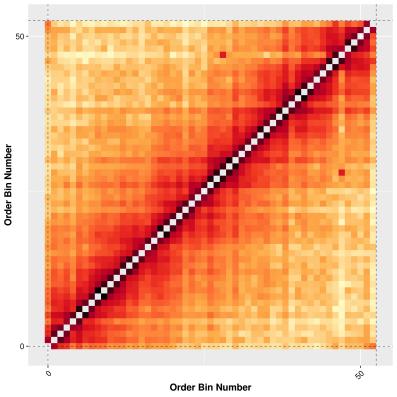  Chr12 |
| 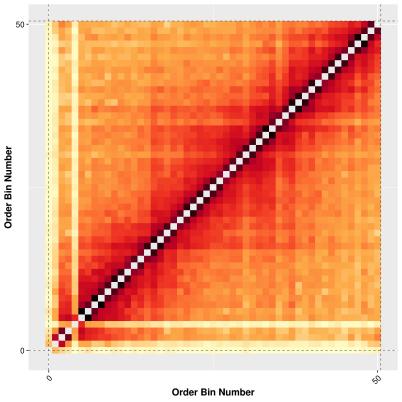  Chr13 | 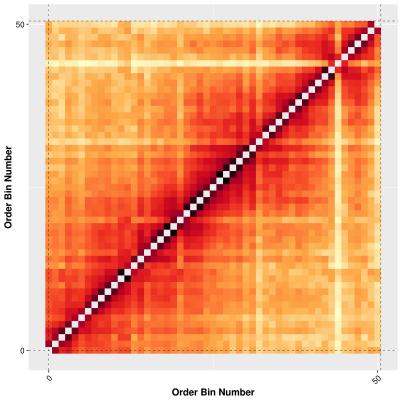  Chr14 | 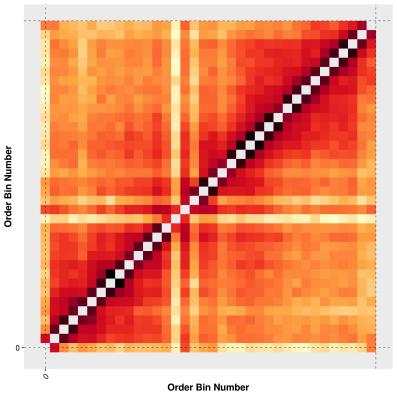  Chr15 |
| 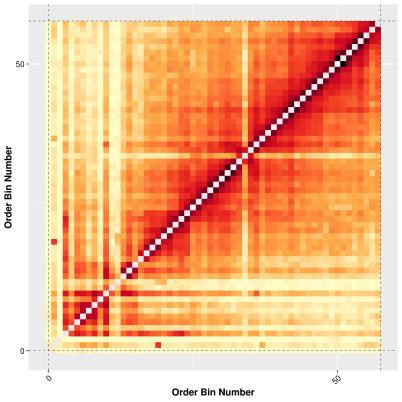  Chr16 | 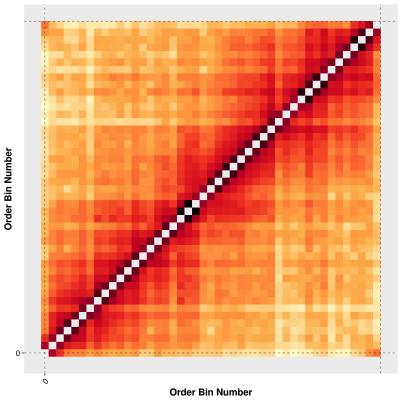  Chr17 | 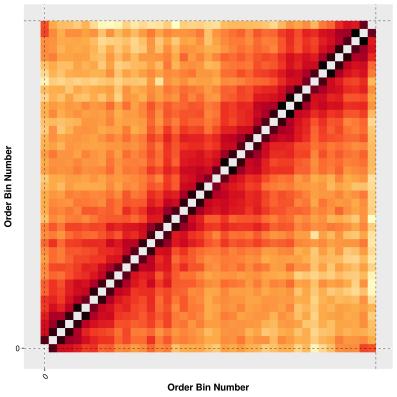  Chr18 |
| 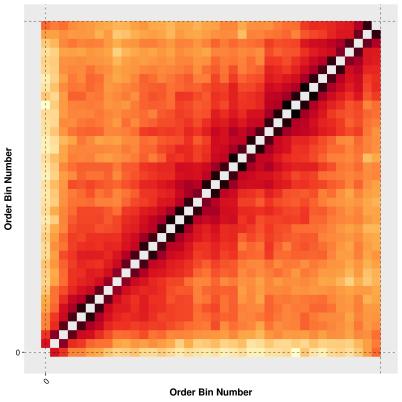  Chr19 | 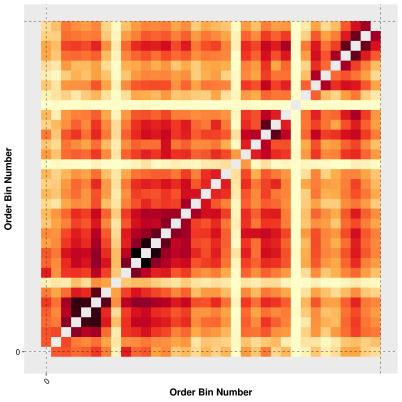  Chr20 | 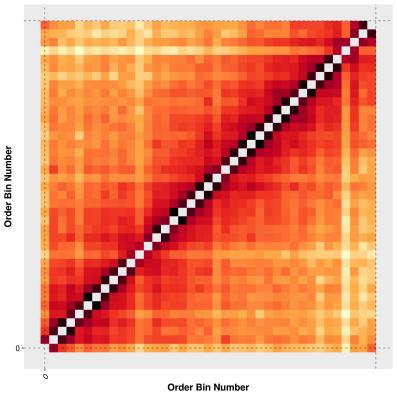  Chr21 |
| 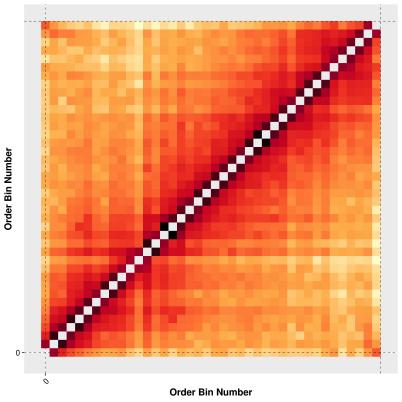  Chr22 | 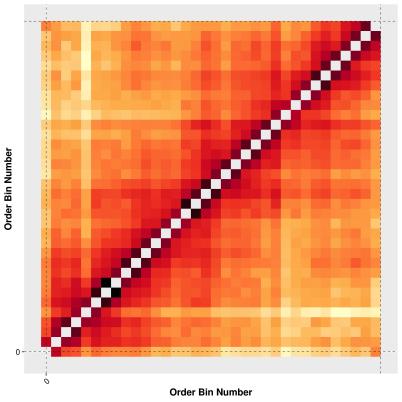  Chr23 | 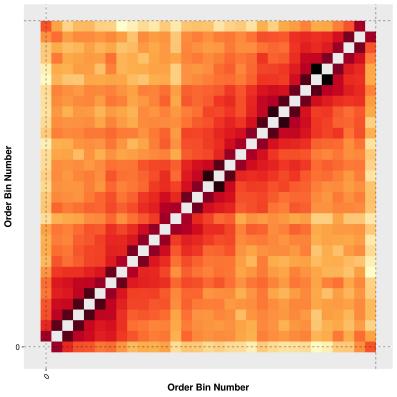  Chr24 |
| 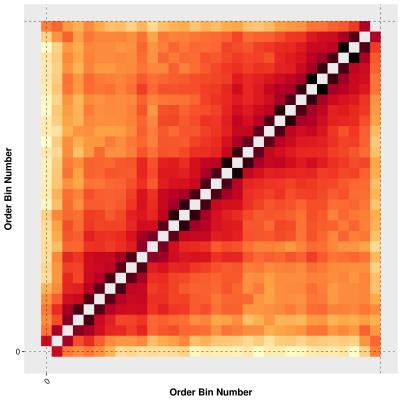  Chr25 | 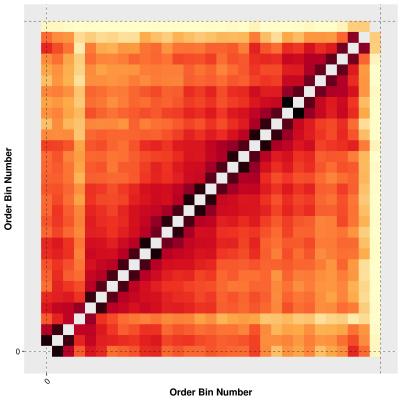  Chr26 | 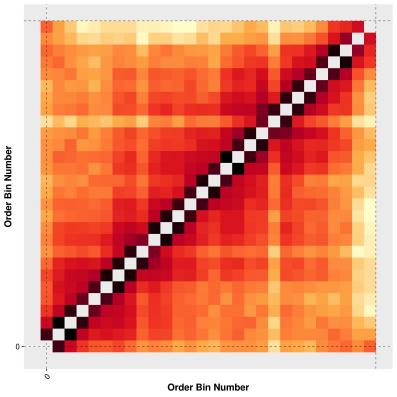  Chr27 |
| 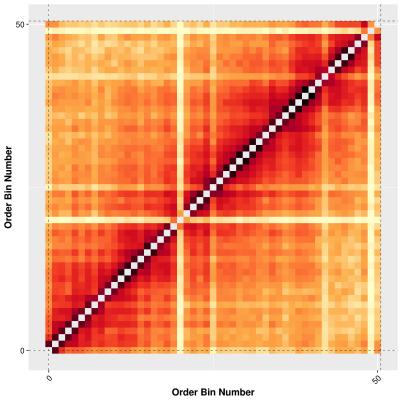  Chr28 | 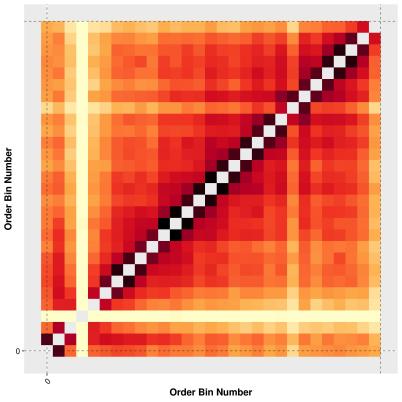  Chr29 | 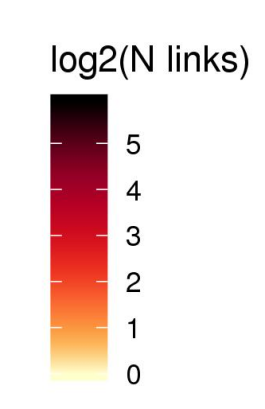 |

**Supplementary Figure S4. Heatmap of each *K.myosuroides* pseudochromosomes** Hi-C interactions are shown at a resolution of 300 kb

**
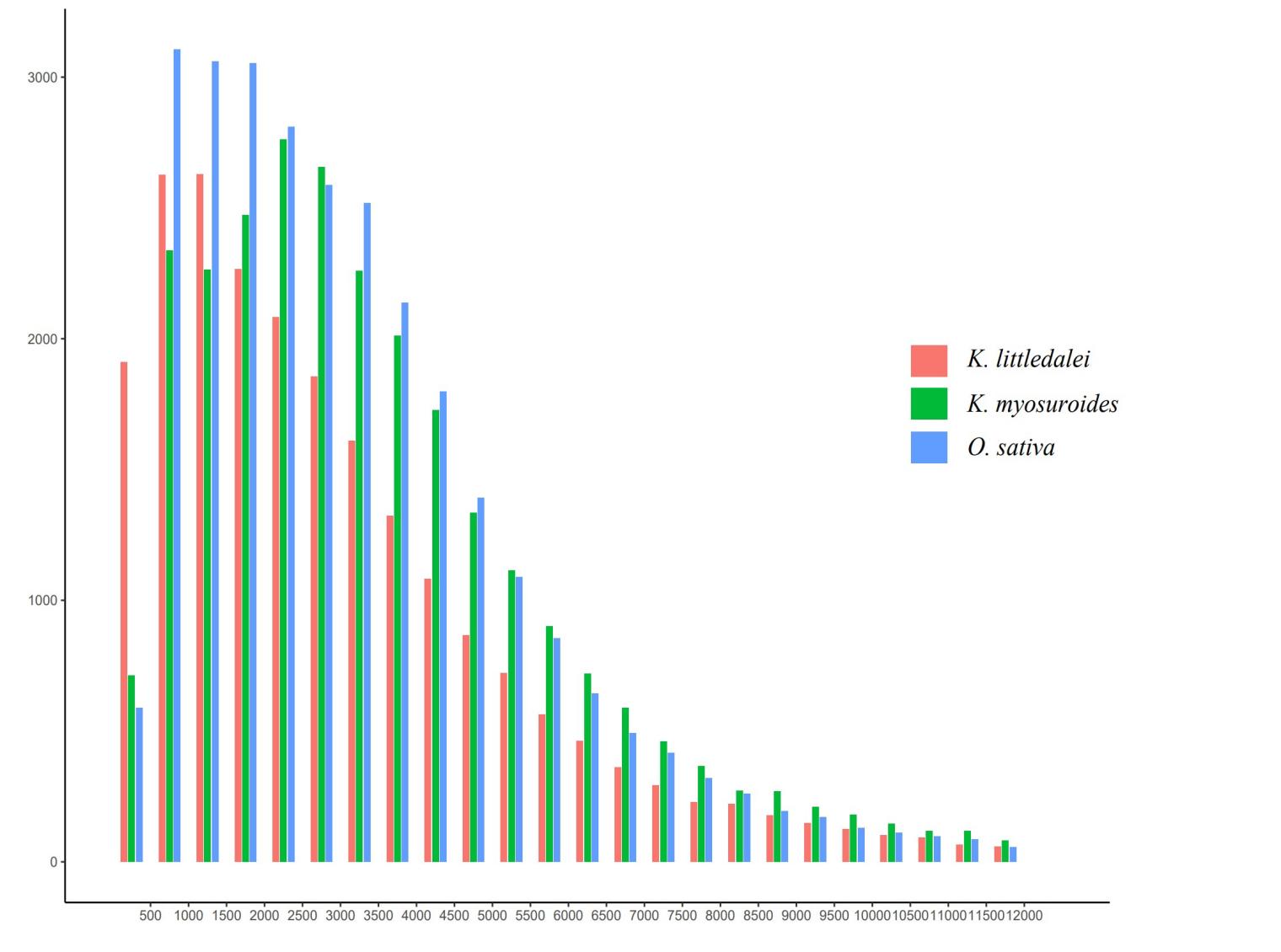
**

**Supplementary Figure S5. Genes Length distribution of *K. myosuroides* and *K. littledalei* with reference to *Oryza sativa*.** X-axis represents the binnd length ranges. Y-axis represents the amount of corresponding genes


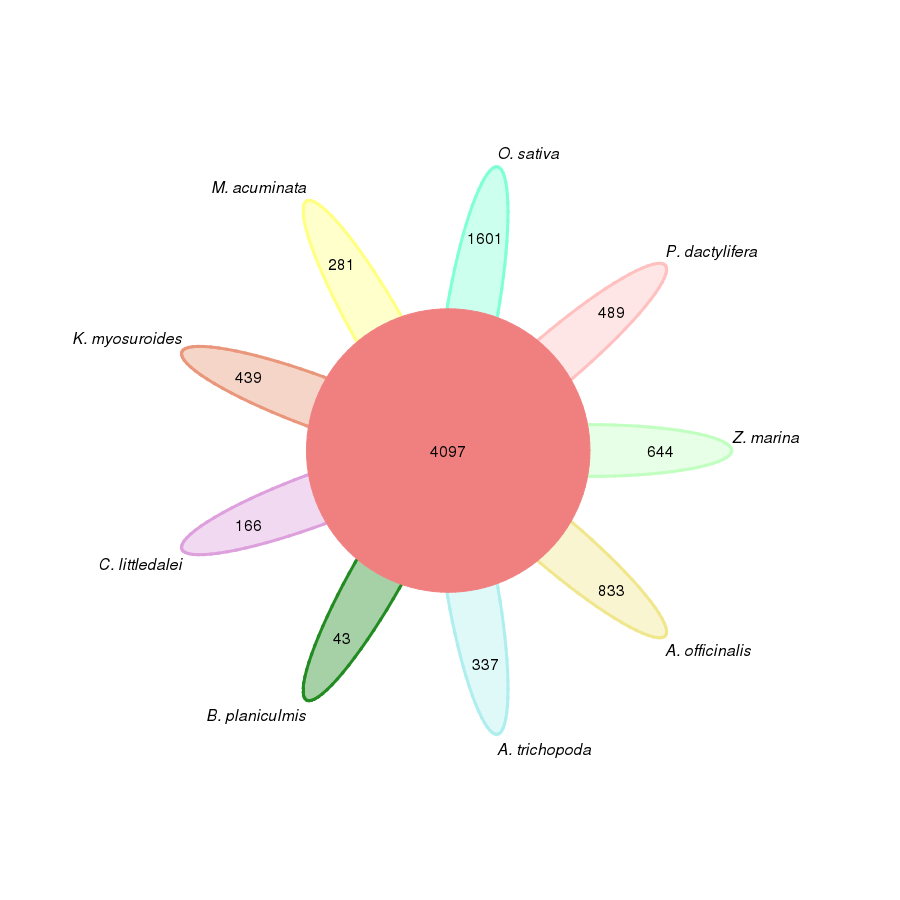


**Supplementary Figure S6. Summary of common and unique gene groups among different species.** Number of common gene groups are shown in the central circle, with number of unique gene groups in the outward tier.


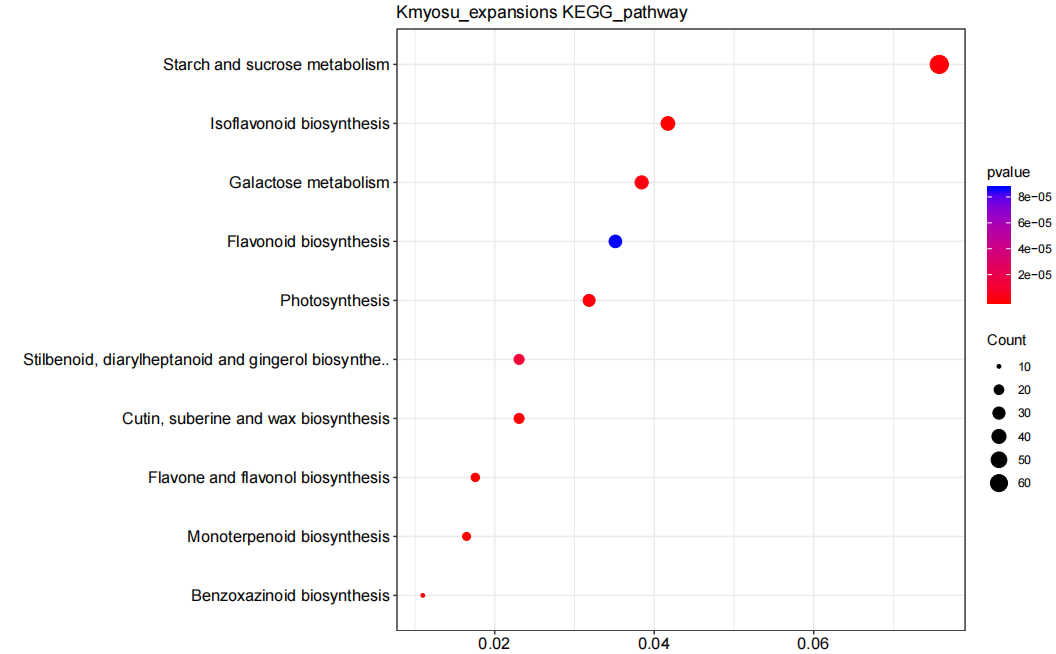


**b**

**a**


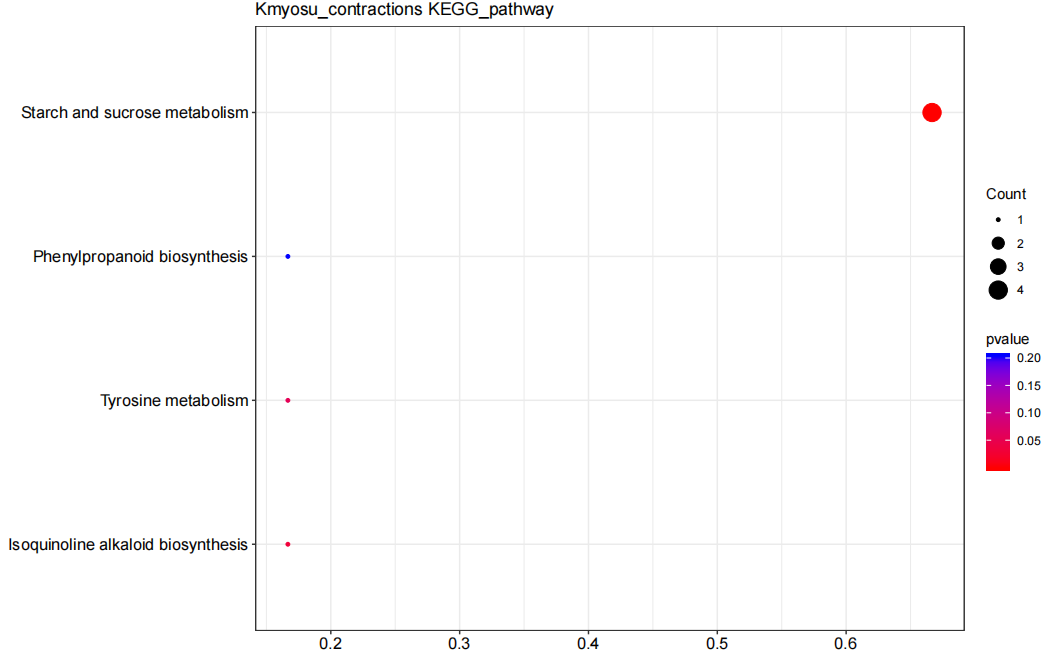


**Supplementary Figure S7. Results of enrichment analysis of (a) expanded gene families and (d) contracted gene families in *K.myosuroides* genome with projections onto KEGG pathways.** Size and color of the symbol are scaled to gene counts and significance level respectively

| 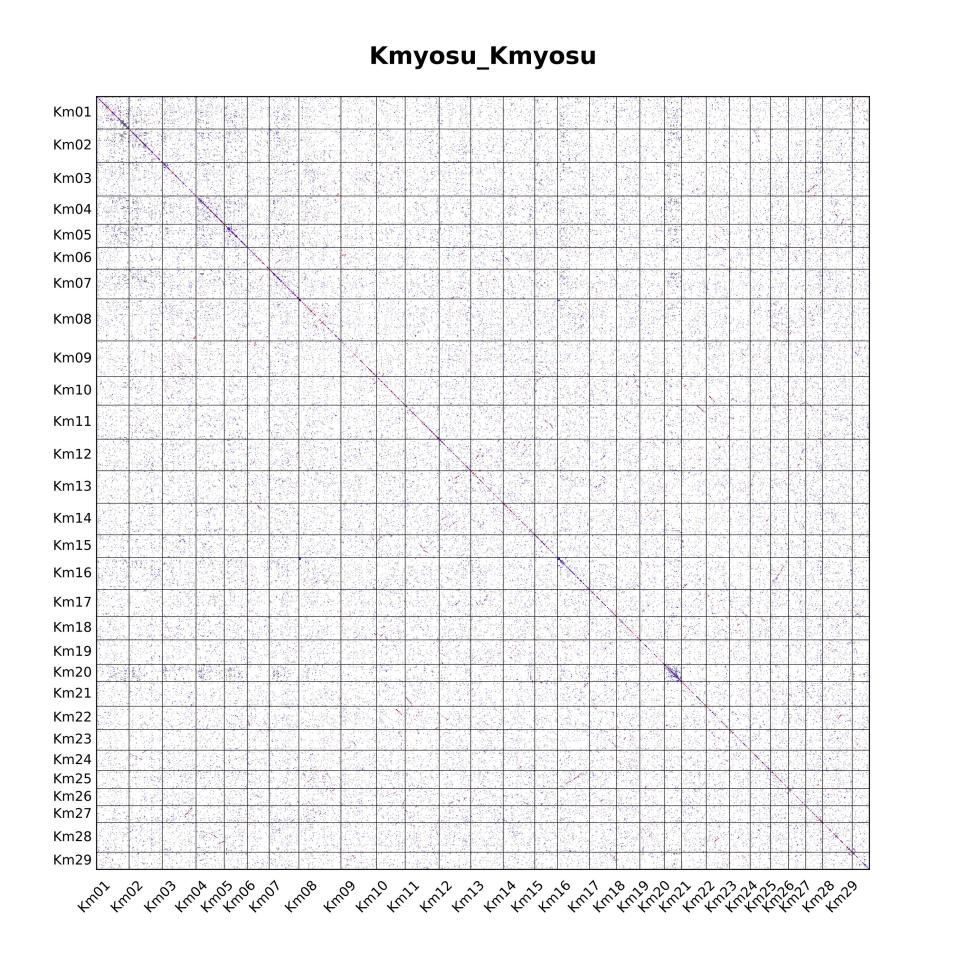  **a** |
| --- |
| 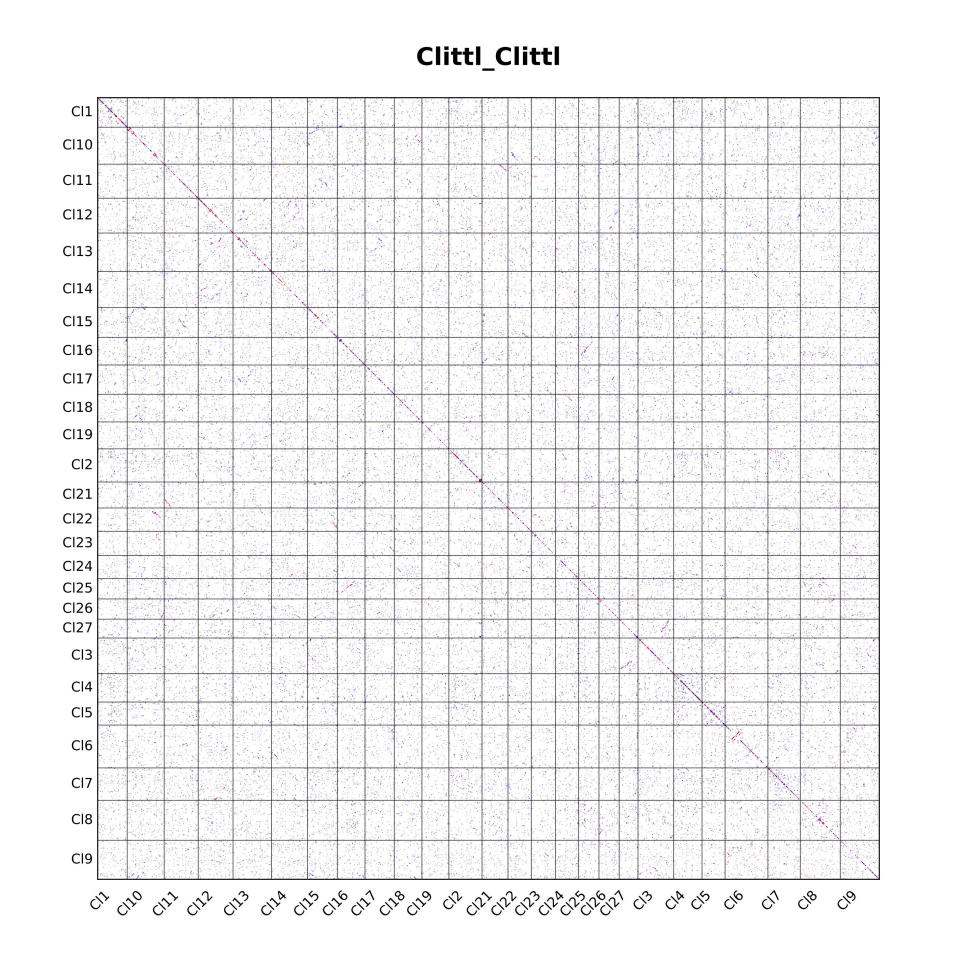  **Supplementary Figure S8. Colinearity dot plot of paralogs of *K. myosuroides*(a) and *K*. *littledalei* (b)** No convincing segments of gene blocks have been detected  **b** |

| 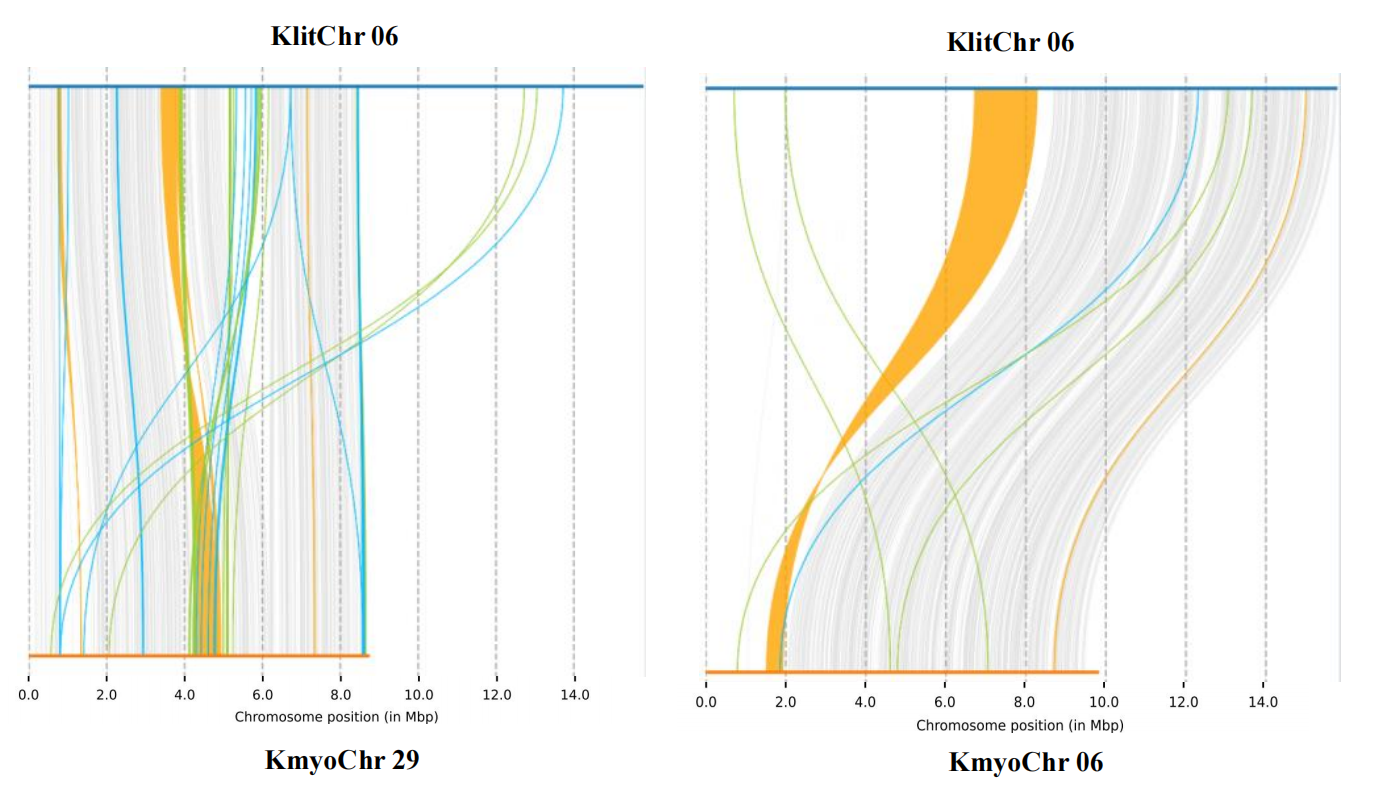 |
| --- |
| 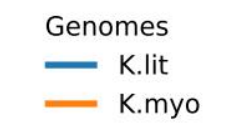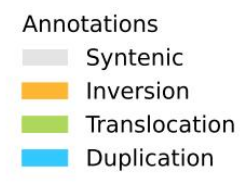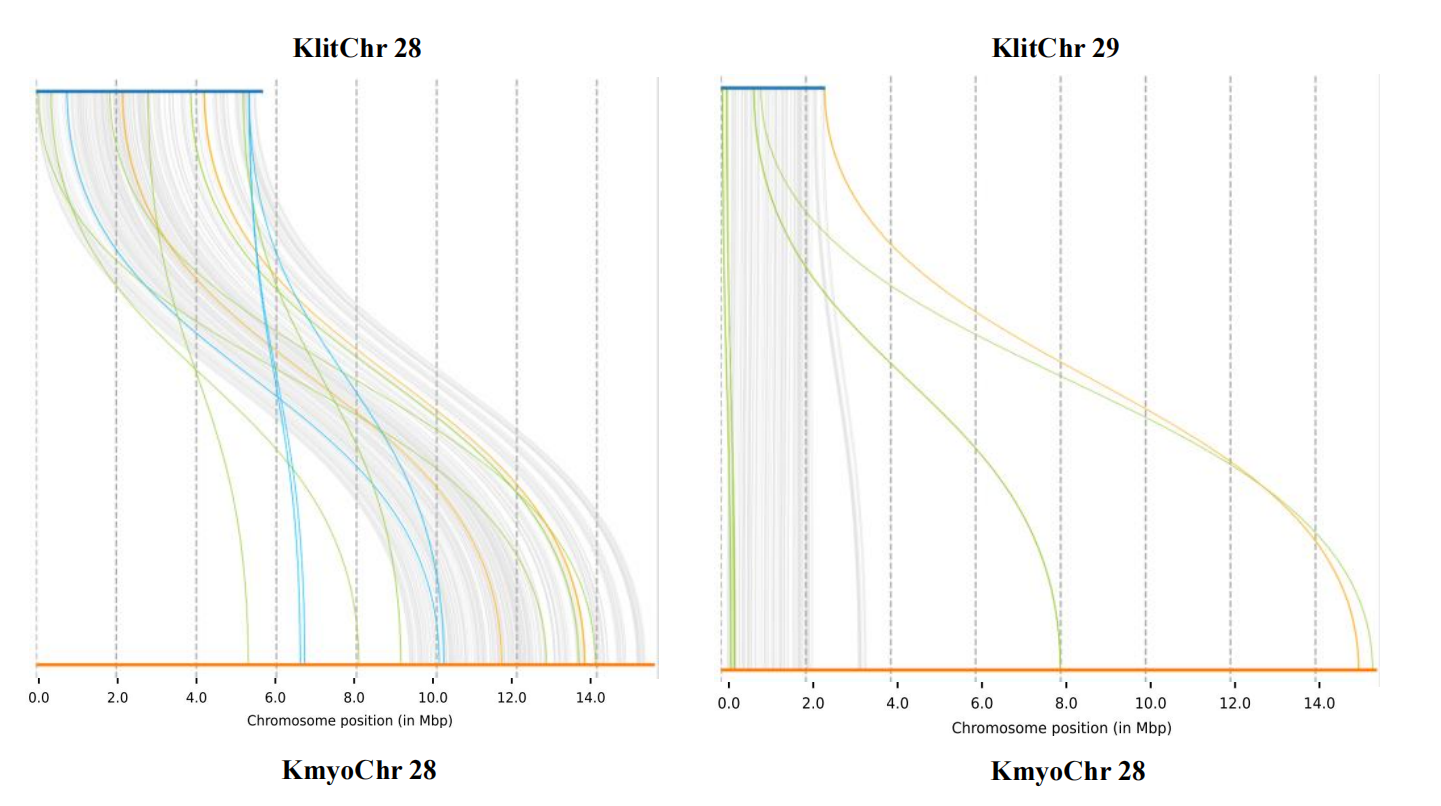 |

**Supplementary Figure S9 Details of synteny and rearrangements among *K. littledalei* chromosome 06, 28,29 and K. myosuroides chromosome 06, 28,29**

**Supplementary Table S1. Summary of sequencing data**

| **Application** | **Library type** | **Platform** | **Total Data (Gb)** | **Reads Number** | **Q30(%)** |
| --- | --- | --- | --- | --- | --- |
| preliminary assemble | Pacbio | Sequel | 21.74 | 1,644,149 | --- |
| Hi-C anchoring | Illumina | NovaSeq 6000 | 53.21 | 177,856,546 | 92.79 |
| survey and mapping back | Illumina | NovaSeq 6000 | 41.84 | 279,246,978 | 91.71 |
| transcriptome | Illumina | NovaSeq 6000 | 12.05 | 40,316,885 | 95.19 |

**Supplementary Table S2.** **Result of flow cytometry of *Kobresia myosuroides***

| **SampleID** | **Reference C-value(Mb)** | **Reference fluorescence density** | **Sample fluorescence density** | **Ratio** | **Estimated C-value(Mb)** |
| --- | --- | --- | --- | --- | --- |
| K.myo-1 | 880 | 22.13 | 10.12 | 0.457 | 402.16 |
| K.myo-2 | 880 | 22.80 | 10.54 | 0.462 | 406.56 |
| K.myo-3 | 880 | 22.43 | 10.57 | 0.471 | 414.48 |
|  |  |  |  |  |  |
| *Average* |  |  |  |  | *407.73* |

**Supplementary Table S3.** **Statistics of the preliminary assembly of *K. myosuroides***

| **Species** | ***Kobresia myosuroides*** |
| --- | --- |
| *Sequence* |  |
| Assembly size (bp) | 413,149,238 |
| GC content (%) | 36.16 |
| Number of contigs | 220 |
| Longest contig (bp) | 19,517,654 |
| Contig N90 size (bp) | 9,325,719 |
| Contig N50 size (bp) | 14,691,126 |
|  |  |
| *BUSCO score* |  |
| Complete BUSCOs (%) | 93.56 |
| Complete & single-copy BUSCOs (%) | 90.77 |
| Complete & duplicated BUSCOs (%) | 2.79 |
| Fragmented BUSCOs (%) | 1.24 |
| Missing BUSCOs (%) | 5.20 |
| Total groups searched | 1614 |

**Supplementary Table S4** **Classification of tandem repeats in the *K. myosuroides* genome.**

| **Type** | **Number** | **Length** | **Rate(%)** |
| --- | --- | --- | --- |
| Microsatellite (1-9 bp units) | 213,467 | 4,361,320 | 1.06 |
| Minisatellite (10-99 bp units) | 139,543 | 16,706,099 | 4.04 |
| Satellite (>=100 bp units) | 11,858 | 7,865,828 | 1.90 |
|  |  |  |  |
| Total | 364,868 | 28,933,247 | 7.00 |

**Supplementary Table S5** **Classification of transposable elements in the *K. myosuroides* genome.**

| **Type** | **Number** | **Length(bp)** | **Rate(%)** |
| --- | --- | --- | --- |
| ***class Ⅰ Retroelement*** | **152,478** | **99,765,425** | **24.15** |
| LINE | 29,558 | 9,389,120 | 2.27 |
| LTR |  |  |  |
| Caulimovirus | 57 | 301,822 | 0.07 |
| Copia | 38,255 | 31,172,558 | 7.55 |
| ERV | 1,009 | 70,633 | 0.02 |
| Gypsy | 22,482 | 35,788,386 | 8.66 |
| Ngaro | 309 | 19,461 | 0.00 |
| Pao | 342 | 91,838 | 0.02 |
| Unknown | 54,884 | 22,131,641 | 5.36 |
| SINE | 5,582 | 799,966 | 0.19 |
|  |  |  |  |
| ***class Ⅱ DNA transposon*** | **251,632** | **85,688,199** | **20.74** |
| Academ | 1 | 73 | 0.00 |
| CACTA | 9,286 | 9,531,334 | 2.31 |
| Crypton | 43 | 1,649 | 0.00 |
| Dada | 175 | 8,333 | 0.00 |
| Ginger | 71 | 3,831 | 0.00 |
| Helitron | 3,100 | 980,699 | 0.24 |
| IS3EU | 87 | 4,213 | 0.00 |
| Kolobok | 326 | 27,028 | 0.01 |
| Maverick | 42 | 3,231 | 0.00 |
| Merlin | 516 | 68,933 | 0.02 |
| Mutator | 16,962 | 15,000,855 | 3.63 |
| P | 121 | 6,765 | 0.00 |
| PIF-Harbinger | 980 | 172,865 | 0.04 |
| PiggyBac | 71 | 2,713 | 0.00 |
| Tc1-Mariner | 1,108 | 267,412 | 0.06 |
| Unknown | 214,665 | 57,620,895 | 13.95 |
| Zisupton | 64 | 3,358 | 0.00 |
| hAT | 4,014 | 1,984,012 | 0.48 |
|  |  |  |  |
| ***Unknown*** | 22 | 1,370 | 0.00 |
|  |  |  |  |
| TOTAL | 404,132 | 185,454,994 | 44.89 |

**Supplementary Table S6** **Number of detected Non-coding RNA and PseudoGene in the genome of *K. myosuroides***

| **Type** | **Number** |
| --- | --- |
| rRNA | 1,616 |
| tRNA | 813 |
| miRNA | 125 |
| snRNA | 58 |
| snoRNA | 24 |
| pseudogene | 116 |

**Supplementary Table S7** **Assessment of assembly quality through mapping back rates and coverage score of both kinds of reads**

|  |  | **Illumina reads** | **Pacbio reads** |
| --- | --- | --- | --- |
| mapping | Total number of reads | 279,246,978 | 1,644,149 |
|  | number of mapped reads | 271,981,896 | 1,594,716 |
|  | Mapping rate (%) | 97.4 | 96.99 |
|  |  |  |  |
| coverage & depth | Coverage (%) | 98.78 | 99.96 |
|  | Coverage at least 5× (%) | 98.61 | 98.97 |
|  | Coverage at least 10× (%) | 98.51 | 98.54 |
|  | Coverage at least 20× (%) | 98.2 | 96.94 |
|  | Average depth | 91 | 49 |

**Supplementary Table S8** **Summary of *K. myosuroides* pseudochromosomes**

| **Chromosome** | **Length (bp)** | **Number of contigs** | **Number of genes** |
| --- | --- | --- | --- |
| Chr01 | 19,009,490 | 7 | 1,070 |
| Chr02 | 18,064,619 | 4 | 1,097 |
| Chr03 | 18,390,026 | 1 | 1,112 |
| Chr04 | 16,825,936 | 3 | 932 |
| Chr05 | 14,691,126 | 3 | 760 |
| Chr06 | 9,830,921 | 3 | 720 |
| Chr07 | 15,824,569 | 3 | 981 |
| Chr08 | 19,427,865 | 3 | 1,386 |
| Chr09 | 17,588,163 | 1 | 1,172 |
| Chr10 | 14,440,547 | 2 | 947 |
| Chr11 | 17,283,893 | 3 | 1,122 |
| Chr12 | 15,971,691 | 1 | 1,042 |
| Chr13 | 15,427,201 | 2 | 1,070 |
| Chr14 | 15,465,566 | 3 | 1,040 |
| Chr15 | 10,820,603 | 2 | 752 |
| Chr16 | 17,403,476 | 4 | 1,054 |
| Chr17 | 13,773,975 | 3 | 884 |
| Chr18 | 12,338,714 | 2 | 776 |
| Chr19 | 11,476,082 | 2 | 807 |
| Chr20 | 10,448,523 | 5 | 570 |
| Chr21 | 11,759,443 | 1 | 813 |
| Chr22 | 12,024,848 | 2 | 761 |
| Chr23 | 10,447,600 | 2 | 687 |
| Chr24 | 9,428,833 | 1 | 673 |
| Chr25 | 9,755,249 | 1 | 593 |
| Chr26 | 9,325,719 | 2 | 566 |
| Chr27 | 8,548,899 | 2 | 558 |
| Chr28 | 15,447,987 | 1 | 975 |
| Chr29 | 8,755,963 | 1 | 567 |
|  |  |  |  |
| Average(±SD) | 13,793,018 (±3,400,343) | 2 (±1) | 879 (±214) |
| Total | 399,997,527 | 70 | 25,487 |

**Supplementary Table S9 List of candidate genes positively selected for or expanded in *K. myosuroides* genome with verified protein product in Swissprot database.**

| **Candidate genes** | **EntryID** | **Protein name** |
| --- | --- | --- |
| Kmy18G002750 | Q8H0T5 | Ethylene-responsive transcription factor ERF073 (AtERF73) (Protein HYPOXIA RESPONSIVE ERF 1) |
| Kmy05G001150 | Q8H0T5 | Ethylene-responsive transcription factor ERF073 (AtERF73) (Protein HYPOXIA RESPONSIVE ERF 1) |
| Kmy11G002490 | Q69QQ6 | Heat shock protein 81-2 (HSP81-2) (Heat shock protein 90) |
| Kmy11G002540 | Q69QQ6 | Heat shock protein 81-2 (HSP81-2) (Heat shock protein 90) |
| Kmy05G006060 | F4HQD4 | Heat shock 70 kDa protein 15 (Heat shock protein 70-15) (AtHsp70-15) |
| Kmy05G006160 | F4HQD4 | Heat shock 70 kDa protein 15 (Heat shock protein 70-15) (AtHsp70-15) |
| Kmy05G006130 | F4HQD4 | Heat shock 70 kDa protein 15 (Heat shock protein 70-15) (AtHsp70-15) |
| Kmy05G006070 | F4HQD4 | Heat shock 70 kDa protein 15 (Heat shock protein 70-15) (AtHsp70-15) |
| Kmy28G001880 | Q9CAQ8 | Replication factor C subunit 5 (AtRFC5) (Activator 1 subunit 5) (Protein EMBRYO DEFECTIVE 2810) |
| Kmy12G007540 | Q9CAQ8 | Replication factor C subunit 5 (AtRFC5) (Activator 1 subunit 5) (Protein EMBRYO DEFECTIVE 2810) |
| Kmy02G003000 | Q5XLY0 | Putative anthocyanidin reductase (GbANR) (EC 1.3.1.-) |
| Kmy02G003020 | Q5XLY0 | Putative anthocyanidin reductase (GbANR) (EC 1.3.1.-) |
| Kmy03G005560 | Q5XLY0 | Putative anthocyanidin reductase (GbANR) (EC 1.3.1.-) |
| Kmy13G000030 | Q5XLY0 | Putative anthocyanidin reductase (GbANR) (EC 1.3.1.-) |
| Kmy28G009750 | Q5XLY0 | Putative anthocyanidin reductase (GbANR) (EC 1.3.1.-) |
| Kmy29G000610 | Q5XLY0 | Putative anthocyanidin reductase (GbANR) (EC 1.3.1.-) |
| Kmy01G004350 | P26413 | Heat shock 70 kDa protein |
| Kmy01G004360 | P11143 | Heat shock 70 kDa protein |
| Kmy05G006140 | P26413 | Heat shock 70 kDa protein |
| Kmy08G003570 | P26413 | Heat shock 70 kDa protein |
| Kmy08G003580 | P26413 | Heat shock 70 kDa protein |
| Kmy08G003590 | P26413 | Heat shock 70 kDa protein |
| Kmy08G003610 | P26413 | Heat shock 70 kDa protein |
| Kmy08G003620 | P11143 | Heat shock 70 kDa protein |
| Kmy08G003630 | P26413 | Heat shock 70 kDa protein |
| Kmy08G003640 | P26413 | Heat shock 70 kDa protein |
| Kmy08G003650 | P26413 | Heat shock 70 kDa protein |
| Kmy24G004060 | P01104 | Transforming protein Myb |
| Kmy24G004080 | P01104 | Transforming protein Myb |
| Kmy24G004160 | P01104 | Transforming protein Myb |
| Kmy05G006260 | P25096 | Protein P21 |
| Kmy10G005960 | P25096 | Protein P21 |
| Kmy10G005970 | P25096 | Protein P21 |
| Kmy01G005280 | G7JMM0 | GRAS family protein RAD1 (Protein REQUIRED FOR ARBUSCULE DEVELOPMENT 1) (MtRAD1) |
| Kmy01G005290 | A0A1B1WAJ0 | GRAS family protein RAD1 (Protein REQUIRED FOR ARBUSCULE DEVELOPMENT 1) (LjRAD1) |
| Kmy03G002930 | A0A1B1WAJ0 | GRAS family protein RAD1 (Protein REQUIRED FOR ARBUSCULE DEVELOPMENT 1) (LjRAD1) |
| Kmy03G003370 | G7JMM0 | GRAS family protein RAD1 (Protein REQUIRED FOR ARBUSCULE DEVELOPMENT 1) (MtRAD1) |
| Kmy05G001040 | G7JMM0 | GRAS family protein RAD1 (Protein REQUIRED FOR ARBUSCULE DEVELOPMENT 1) (MtRAD1) |
| Kmy10G005890 | G7JMM0 | GRAS family protein RAD1 (Protein REQUIRED FOR ARBUSCULE DEVELOPMENT 1) (MtRAD1) |
| Kmy10G005900 | G7JMM0 | GRAS family protein RAD1 (Protein REQUIRED FOR ARBUSCULE DEVELOPMENT 1) (MtRAD1) |
| Kmy10G005910 | G7JMM0 | GRAS family protein RAD1 (Protein REQUIRED FOR ARBUSCULE DEVELOPMENT 1) (MtRAD1) |
| Kmy12G010290 | G7JMM0 | GRAS family protein RAD1 (Protein REQUIRED FOR ARBUSCULE DEVELOPMENT 1) (MtRAD1) |
| Kmy13G003350 | G7JMM0 | GRAS family protein RAD1 (Protein REQUIRED FOR ARBUSCULE DEVELOPMENT 1) (MtRAD1) |
| Kmy13G003370 | A0A1B1WAJ0 | GRAS family protein RAD1 (Protein REQUIRED FOR ARBUSCULE DEVELOPMENT 1) (LjRAD1) |
| Kmy16G004820 | A0A1B1WAJ0 | GRAS family protein RAD1 (Protein REQUIRED FOR ARBUSCULE DEVELOPMENT 1) (LjRAD1) |
| Kmy16G004830 | G7JMM0 | GRAS family protein RAD1 (Protein REQUIRED FOR ARBUSCULE DEVELOPMENT 1) (MtRAD1) |
| Kmy29G002650 | A0A1B1WAJ0 | GRAS family protein RAD1 (Protein REQUIRED FOR ARBUSCULE DEVELOPMENT 1) (LjRAD1) |
| Kmy01G002860 | G7JMM0 | GRAS family protein RAD1 (Protein REQUIRED FOR ARBUSCULE DEVELOPMENT 1) (MtRAD1) |
| Kmy04G003330 | G7JMM0 | GRAS family protein RAD1 (Protein REQUIRED FOR ARBUSCULE DEVELOPMENT 1) (MtRAD1) |
| Kmy08G007090 | A0A1B1WAJ0 | GRAS family protein RAD1 (Protein REQUIRED FOR ARBUSCULE DEVELOPMENT 1) (LjRAD1) |
| Kmy08G007100 | A0A1B1WAJ0 | GRAS family protein RAD1 (Protein REQUIRED FOR ARBUSCULE DEVELOPMENT 1) (LjRAD1) |
| Kmy16G004860 | G7JMM0 | GRAS family protein RAD1 (Protein REQUIRED FOR ARBUSCULE DEVELOPMENT 1) (MtRAD1) |
| Kmy16G004870 | A0A1B1WAJ0 | GRAS family protein RAD1 (Protein REQUIRED FOR ARBUSCULE DEVELOPMENT 1) (LjRAD1) |
| Kmy02G003270 | Q6F4N5 | Aspartyl protease 25 (EC 3.4.23.-) (Protein RICE ANTHER DOWN-REGULATED BY CHILLING 1) |
| Kmy13G008920 | Q6F4N5 | Aspartyl protease 25 (EC 3.4.23.-) (Protein RICE ANTHER DOWN-REGULATED BY CHILLING 1) |
| Kmy02G002670 | Q9SRX6 | Late embryogenis abundant protein 2 |
| Kmy02G002700 | Q9SRX6 | Late embryogenis abundant protein 2 |
| Kmy05G002950 | P46522 | Late embryogenesis abundant protein Lea5-D |
| Kmy07G001380 | P46522 | Late embryogenesis abundant protein Lea5-D |
| Kmy16G008590 | P0C5A4 | Late embryogenesis abundant protein 19 (OsLEA19) (Late embryogenesis abundant protein, group 3) (LEA-3) (OsLEA3-1) |
| Kmy16G008610 | Q03968 | Late embryogenesis abundant protein, group 3 (LEA) (PMA2005) |
| Kmy16G008620 | Q42376 | Late embryogenesis abundant protein, group 3 (LEA) |
| Kmy16G008630 | Q42376 | Late embryogenesis abundant protein, group 3 (LEA) |
| Kmy17G005940 | L7X3S1 | Methyltetrahydroprotoberberine 14-monooxygenase (EC 1.14.14.97) ((S)-cis-N-methylstylopine 14-hydroxylase) ((S)-cis-N-methyltetrahydroprotoberberine-14-hydroxylase) (Methyltetrahydroprotoberberine 14-hydroxylase) (N-methylstylopine hydroxylase) (MSH) |
| Kmy20G002400 | L7X3S1 | Methyltetrahydroprotoberberine 14-monooxygenase (EC 1.14.14.97) ((S)-cis-N-methylstylopine 14-hydroxylase) ((S)-cis-N-methyltetrahydroprotoberberine-14-hydroxylase) (Methyltetrahydroprotoberberine 14-hydroxylase) (N-methylstylopine hydroxylase) (MSH) |
| Kmy20G002410 | L7X3S1 | Methyltetrahydroprotoberberine 14-monooxygenase (EC 1.14.14.97) ((S)-cis-N-methylstylopine 14-hydroxylase) ((S)-cis-N-methyltetrahydroprotoberberine-14-hydroxylase) (Methyltetrahydroprotoberberine 14-hydroxylase) (N-methylstylopine hydroxylase) (MSH) |
| Kmy20G002430 | L7X3S1 | Methyltetrahydroprotoberberine 14-monooxygenase (EC 1.14.14.97) ((S)-cis-N-methylstylopine 14-hydroxylase) ((S)-cis-N-methyltetrahydroprotoberberine-14-hydroxylase) (Methyltetrahydroprotoberberine 14-hydroxylase) (N-methylstylopine hydroxylase) (MSH) |
| Kmy24G004120 | F4IRB4 | Transcription factor MYB88 (Myb-related protein 88) (AtMYB88) |
| Kmy24G004140 | F4IRB4 | Transcription factor MYB88 (Myb-related protein 88) (AtMYB88) |
| Kmy21G007130 | O49687 | Transcription factor MYC4 (AtMYC4) (Basic helix-loop-helix protein 4) (AtbHLH4) (bHLH 4) (Transcription factor EN 37) (bHLH transcription factor bHLH004) |
| Kmy28G006850 | Q9LSE2 | Transcription factor ICE1 (Basic helix-loop-helix protein 116) (AtbHLH116) (bHLH 116) (Inducer of CBF expression 1) (Transcription factor EN 45) (Transcription factor SCREAM) (bHLH transcription factor bHLH116) |
| Kmy05G003180 | Q9LSE2 | Transcription factor ICE1 (Basic helix-loop-helix protein 116) (AtbHLH116) (bHLH 116) (Inducer of CBF expression 1) (Transcription factor EN 45) (Transcription factor SCREAM) (bHLH transcription factor bHLH116) |
| Kmy25G001850 | Q84LK0 | DNA mismatch repair protein MSH1, mitochondrial (AtMSH1) (MutS protein homolog 1) (Protein CHLOROPLAST MUTATOR) |
| Kmy24G001160 | Q9LEM8 | PsbD mRNA maturation factor Nac2, chloroplastic |
| Kmy03G010960 | A0A1P8ASY1 | DNA replication ATP-dependent helicase/nuclease JHS1 (Protein EMBRYO DEFECTIVE 2411) (Protein JING HE SHENG 1) [Includes: DNA replication nuclease JHS1 (EC 3.1.-.-); DNA replication ATP-dependent helicase JHS1 (EC 3.6.4.12)] |
| Kmy05G000610 | F4I2N7 | Receptor-like protein kinase 7 (EC 2.7.11.1) |
| Kmy05G000960 | F4I2N7 | Receptor-like protein kinase 7 (EC 2.7.11.1) |
| Kmy22G004230 | F4I2N7 | Receptor-like protein kinase 7 (EC 2.7.11.1) |
| Kmy03G000560 | F4I2N7 | Receptor-like protein kinase 7 (EC 2.7.11.1) |
| Kmy07G001890 | F4I2N7 | Receptor-like protein kinase 7 (EC 2.7.11.1) |
